# Host–Viral Interactions Revealed among Shared Transcriptomics Signatures of ARDS and Thrombosis: A Clue into COVID-19 Pathogenesis

**DOI:** 10.1055/s-0040-1721706

**Published:** 2020-12-17

**Authors:** Aastha Mishra, Shankar Chanchal, Mohammad Z. Ashraf

**Affiliations:** 1 Department of Biotechnology, Faculty of Natural Sciences, Jamia Millia Islamia, New Delhi, India

**Keywords:** COVID-19, SARS-COV-2, ARDS, thrombosis, inflammation

## Abstract

Severe novel corona virus disease 2019 (COVID-19) infection is associated with a considerable activation of coagulation pathways, endothelial damage, and subsequent thrombotic microvascular injuries. These consistent observations may have serious implications for the treatment and management of this highly pathogenic disease. As a consequence, the anticoagulant therapeutic strategies, such as low molecular weight heparin, have shown some encouraging results. Cytokine burst leading to sepsis which is one of the primary reasons for acute respiratory distress syndrome (ARDS) drive that could be worsened with the accumulation of coagulation factors in the lungs of COVID-19 patients. However, the obscurity of this syndrome remains a hurdle in making decisive treatment choices. Therefore, an attempt to characterize shared biological mechanisms between ARDS and thrombosis using comprehensive transcriptomics meta-analysis is made. We conducted an integrated gene expression meta-analysis of two independently publicly available datasets of ARDS and venous thromboembolism (VTE). Datasets GSE76293 and GSE19151 derived from National Centre for Biotechnology Information–Gene Expression Omnibus (NCBI-GEO) database were used for ARDS and VTE, respectively. Integrative meta-analysis of expression data (INMEX) tool preprocessed the datasets and effect size combination with random effect modeling was used for obtaining differentially expressed genes (DEGs). Network construction was done for hub genes and pathway enrichment analysis. Our meta-analysis identified a total of 1,878 significant DEGs among the datasets, which when subjected to enrichment analysis suggested inflammation–coagulation–hypoxemia convolutions in COVID-19 pathogenesis. The top hub genes of our study such as tumor protein 53 (TP53), lysine acetyltransferase 2B (KAT2B), DExH-box helicase 9 (DHX9), REL-associated protein (RELA), RING-box protein 1 (RBX1), and proteasome 20S subunit beta 2 (PSMB2) gave insights into the genes known to be participating in the host–virus interactions that could pave the way to understand the various strategies deployed by the virus to improve its replication and spreading.

## Introduction

The comprehensive understanding of severe acute respiratory syndrome-coronavirus-2 (SARS-CoV-2) pathogenesis, responsible for the novel coronavirus disease 2019 (COVID-19) outbreak, is not only important for its management but will also take cognizance of the other members of the coronavirus family that have the potential of such outbreaks in future. Acute respiratory distress syndrome (ARDS), as a consequence of acute inflammation, often leading to a reduction of lung function is being widely responsible for the critical illness in the SARS-CoV-2 infected patient. 1 Endothelial damage, activation of coagulation pathways and subsequent generalized thrombotic microvascular injuries could also be observed in the critical COVID-19 patients. 2 3 This abnormality in blood coagulation proteins increases the propensities of venous thromboembolism (VTE), a secondary complication to several diseases including cancers and viral infections such as SARS-CoV-2. 4 Consistent observations of ARDS associated with thrombosis in severe COVID-19 patients may have serious implications for treatment and management. As a consequence, the therapeutic strategies targeting these abnormal levels of cytokines and coagulant factors have shown some encouraging results. Low molecular weight heparin (LMWH) or enoxaparin that inhibits activated factor X is now widely recommended as an early anticoagulation therapy unless there is contraindication. 3 5 Its anti-inflammatory properties further make LMWH, a preferred choice in mitigating cytokine storms in COVID-19 patients. 3 5 Cytokine burst leading to sepsis is one of the primary reasons for the ARDS drive that could be worsened with the accumulation of coagulation factors in the lungs. 6 However, the obscurity of ARDS remains a hurdle in making decisive treatment choices for patients. Normally, ARDS is a build-up of noncardiogenic pulmonary edema resulting in hypoxemia in the vital organs of the body. It is a life-threatening condition that is always associated with a heterogeneous mix of other existing health problems such as sepsis. 7 Though ARDS in COVID-19 is received with a debate among the scientific community, 8 9 still its occurrence along with the endothelial injury in the severe COVID-19 patients cannot be overlooked. 10 Perhaps, the endothelial injury as a result of SARS-CoV-2 infection might have a major role in the development of VTE, as well as ARDS. 11 Thus, it becomes pertinent to ascertain the intrinsic factors responsible for ARDS and thrombotic events in the case of COVID-19 as it is clear that these complications coexist relatedly. This study, therefore, tries to understand the link between ARDS and thrombosis through the revelation of shared biological mechanisms. An attempt is made to comprehend the shared transcriptomics signatures among the datasets derived from ARDS and VTE through network-based meta-analysis. This will aid in delineating the deregulated pathways driving COVID-19 pathophysiology that could further help in deriving standard laboratory tests and targeted therapeutic interventions.

## Materials and Methods

### Curation and Identification of Suitable Gene Expression Datasets for Meta-analysis

**Fig. 1:**
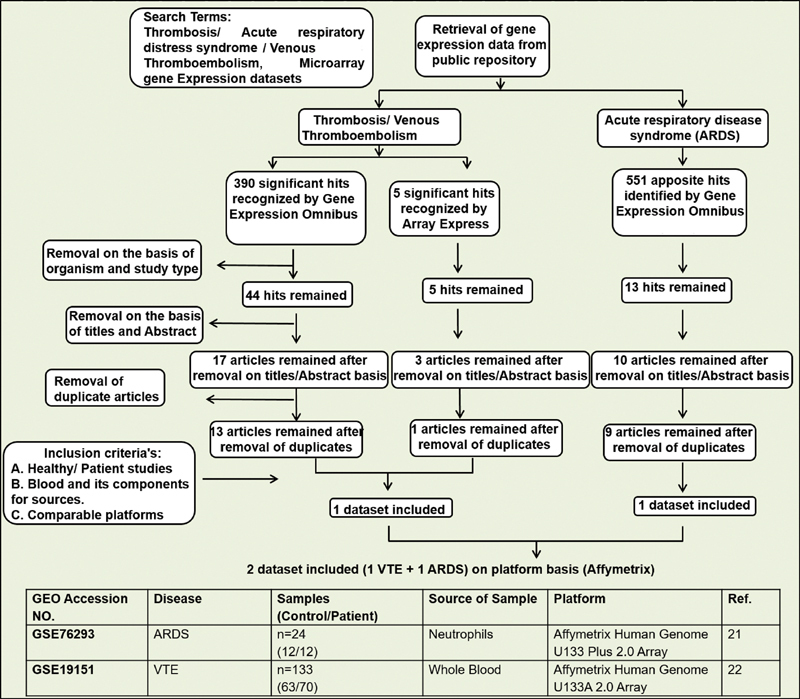
Flow diagram depicting the selection of microarray meta-analysis and characteristics of individual studies included in the study. ARDS, acute respiratory distress syndrome, GEO, gene expression omnibus; VTE, venous thromboembolism.

We conducted an integrated gene expression meta-analysis of the two independently and publicly available datasets for ARDS and VTE. The National Centre for Biotechnology Information–Gene Expression Omnibus (NCBI-GEO) database ( http://www.ncbi.nlm.nih.gov/geo/ ) and European Molecular Biology Laboratory–European Bioinformatics Institute Array Express database ( http://www.ebi.ac.uk/arrayexpress/ ) were mined for microarray-based studies. The following keywords and their combinations were used: “Thrombosis,” “venous thromboembolism,” “ARDS,” “microarray,” “gene expression dataset.” We extracted the following information from each identified study: GEO accession number, sample type, platform, number of controls, and cases along with references. Inclusion criteria were set and strictly followed for dataset selection. The criteria were human case/control study, sample source, platform, and comparable conditions. A Flow diagram depicting the microarray meta-analysis as a selection process of eligible microarray datasets is represented in Fig. 1 .

### Preprocessing Individual Datasets

A web interface for integrative meta-analysis, integrative meta-analysis of expression data (INMEX; http://www.networkanalyst.ca/faces/home.xhtml ) tool was used for preprocessing of datasets using log2-transformation with quantile autoscaling. 12 The data were annotated after converting the gene and probe IDs to the corresponding Entrez IDs. Before performing the meta-analysis, the batch effect correction option was applied to reduce the potential study-specific batch effects heterogeneity using the combat procedure of the INMEX tool. The batch effect removal is needed for the integrative analysis and reducing contradictory factors due to nonbiological variation. Principal component analysis (PCA) was performed to visualize the sample clustering patterns before and after applying the ComBat procedure. 13 The ComBat procedures stabilize the expression ratios of genes with too high or too low ratios using the Empirical Bayes methods and stabilize the individual gene variances by shrinking variances across all the other genes.

### Identification of Shared Differentially Expressed Genes

Differential expression analysis for each dataset was performed with INMEX independently using adjusted *p* < 0.05, based on the false discovery rate using the Benjamini–Hochberg procedure and moderated *t* -test based on the Limma algorithm. 14 For the meta-analysis, data integrity was checked to confirm the consistency and completeness of class labels across all the datasets. The differential expression meta-analysis across the diseases and healthy controls was performed by the random effects model (REM) based on combining the effect sizes (ESs) or changes of gene expression from the different studies and obtaining an overall mean with a significance level of *p* < 0.05. 15 The REM was chosen over the Fixed Effect Model to avoid significant cross-study heterogeneities based on the Cochran's Q-test. 16

### Pathway Analysis Using Kobas3.0

To further explore the biological functions of the shared DEGs of ARDS and VTE, the significant pathway analysis was performed by using the online web site of Kobas3.0 ( http://kobas.cbi.pku.edu.cn/kobas3/?t=1 ) under the function of “Gene list Enrichment” including gene ontology, the Kyoto Encyclopedia of Genes and Genomes (KEGG), Reactome, PANTHER, and few other pathway analyses. 17 It uses a hypergeometric test/Fisher's exact test as a statistical test method with Benjamini and Hochberg as false discovery rate (FDR) correction method.

### Network-Based Hub Gene Analysis and Visual Analytics

Network-based meta-analysis using NetworkAnalyst/INMEX was implemented to generate a protein–protein interaction (PPI) network. A complete list of DEGs was uploaded into the web-based server of NetworkAnalyst. However, the network construction was restricted to contain only the original seed proteins by selecting the zero-order interactors to avoid “hairball effect” and to allow the proper visualization of the interaction network. The obtained result was fed into the Cytoscape tool, which was used for the hub gene analysis showing detailed information on nodes within the current network, including degree, betweenness centrality, and expression. 18 The degree was defined as the number of connections to the other nodes. The betweenness centrality was the number of shortest paths going through a node. 19

### Functional Analysis of Shared DEGs between ARDS and Thrombosis

ClueGO, a Cytoscape plug-in, was utilized to further gain insights into a functionally grouped network of an enriched biological pathway on the shared DEGs. 20 The zero-order interaction network with 519 nodes was downloaded and fed into the Cytoscape with their expression values and additionally, the names of top 20 hub genes were provided to ClueGo for exploring the enriched pathways and biological terms related to our DEGs/networks. We have specifically selected our top 20 hub genes as these genes have high degree of connectivity in the PPI network for our functional analysis. ClueGO is a user-friendly tool to examine the interrelations of terms and functional groups in biological networks. It allows a variety of flexible adjustments for a profound exploration of gene clusters in biological networks. It visualizes the nonredundant biological terms for large clusters of genes and pathways resulted from functional enrichment analysis in a grouped network. We used enrichment (right-sided) hypergeometric distribution tests. The GO terms and pathways were ranked based on their significance with a cut-off *p* ≤ 0.05, followed by the Bonferroni adjustment for the terms.

### Statistical Analyses

The ES combination using the REM was used for the meta-analysis by a web-based tool, INMEX. DEGs were selected based on the FDR using the Benjamini–Hochberg procedure with an adjusted *p* -value of <0.05. Hypergeometric test (right sided) and Benjamini–Hochberg FDR correction with an adjusted *p* -value of ≤0.05 were used for the functional enrichment analysis.

## Results

### Selection of Eligible Microarray Datasets

A total of two studies met the inclusion criteria and were selected for the meta-analysis. The datasets GSE76293 and GSE19151 covered ARDS 21 and VTE, 22 respectively. Fig. 1 provides the detailed information of each datasets and highlights the disease condition, sample groups, source of samples, microarray platform used, and references of the studies. The datasets included in this meta-analysis were case/control studies with a collective number of 12/12 and 70/63 for ARDS and VTE, respectively. The gene expression of treated samples used in ARDS was excluded from our meta-analysis.

### Reduction of Confounding Factors by ComBat Procedures

**Fig. 2:**
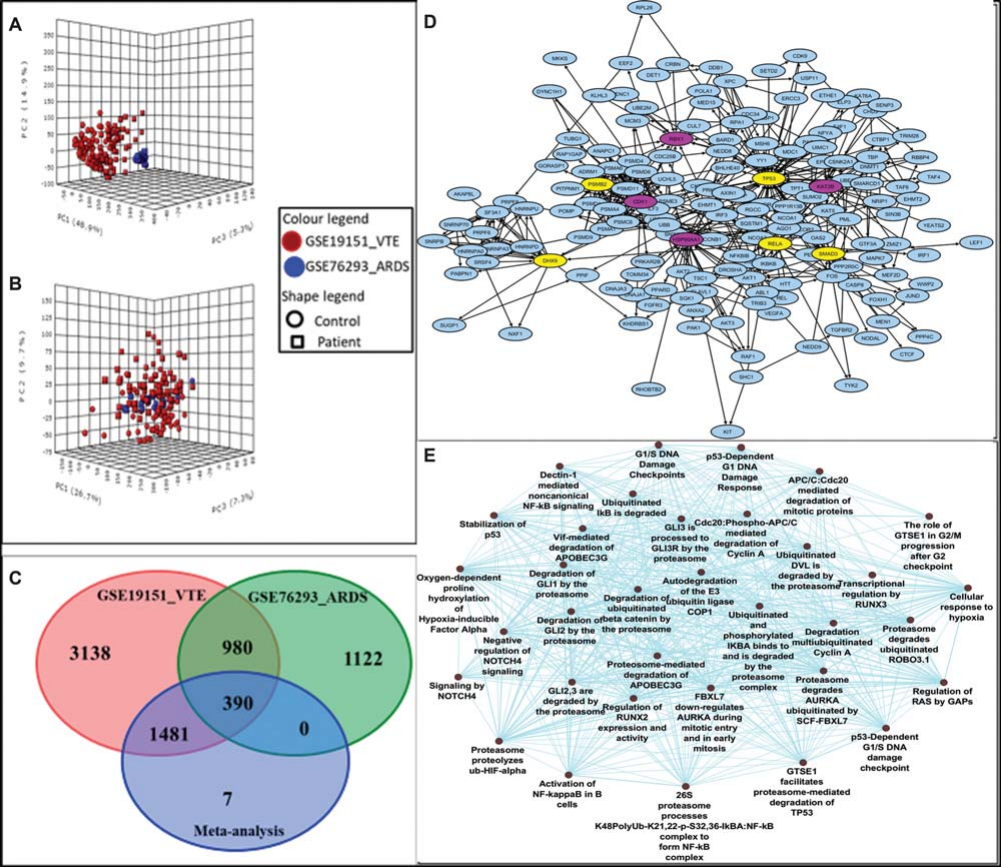
Result illustrations of comprehensive transcriptomics meta-analysis between acute respiratory distress syndrome (ARDS) and venous thromboembolism (VTE) using integrative meta-analysis of expression data (INMEX) tool ( **A** ) Plot of principal component analysis (PCA) as validation tool before batch effect removal and ( **B** ) after batch effect removal (using Combat method). ( **C** ) The Venn diagram comparing differentially expressed genes (DEGs) sets identified by the individual studies and by meta-analysis. The results obtained by the meta-analysis (1,878 DEGs) are compared with DEGs identified by individual study of VTE and ARDS. ( **D** ) Network depicting zero-order interaction of the shared DEGs in between ARDS and VTE datasets. Among the top 20 hub genes, few selected hub genes known to participate in host-viral interaction are shown in purple (overexpressed genes) and yellow (underexpressed gene) colors using Cytoscape. ( **E** ) Overrepresentation of pathways and gene ontology categories in biological networks identified from meta-analysis. Network representations of enriched pathway integrating the Kyoto Encyclopedia of Genes and Genomes (KEGG) and Reactome pathways along with Gene ontology for the selected top 20 hub genes using ClueGO Cytoscape plug-in. Hyper-geometric enrichment distribution tests, with an adjusted *p* -value of ≤0.05, followed by the Bonferroni adjustment for the terms and term groups were selected based on the highest significance.

Before identifying the shared DEGs between ARDS and VTE, the datasets were preprocessed and normalized using the INMEX tool. ComBat procedures took care of reducing the potential study-specific “batch effects.” Fig. 2A visually examines the sample clustering patterns before applying the batch adjustment algorithm using the principal component analysis (PCA). Fig. 2B visualizes the inter mixing of all samples from the datasets after the correction of the batch effect. This demonstrates removing of the confounding factors due to the nonbiological variations and thereby, reducing the potential study-specific “batch effects.”

### Shared Transcriptomics Signature among ARDS and VTE

The implementation of ES and REM statistical analysis of INMEX identified a common transcriptional signature shared between ARDS and VTE. A total of 1,878 DEGs including 736 overexpressed and 1,142 underexpressed genes were identified under the significance threshold of adjusted *p* -value of <0.05. The complete list of significant overexpressed and underexpressed DEGs from the meta-analysis with combined effect size and adjusted *p* < 0.05 is provided in Supplementary Table S1 . Based on the values of combined ES, the basic Leucine Zipper ATF-like transcription factor (BATF), ATP synthase membrane subunit 6.8PL (ATP5MPL), inorganic pyrophosphatase 2 (PPA2), purine nucleoside phosphorylase (PNP), glutathione S-transferase omega-1 (GSTO1), proteasome 20S subunit alpha 6 (PSMA6), transportin 1 (TNPO1), insulin-like growth factor-binding protein 7 (IGFBP7), NADH:ubiquinone oxidoreductase subunit C2 (NDUFC2), and membrane spanning 4-domains A4A (MS4A4A) were among the most overexpressed genes with significant *p* -values. While, nucleoporin 93 (NUP93), cas scaffold protein family member 4 (CASS4), NLR family pyrin domain containing 1 (NLRP1), WW domain binding protein 11 (WBP11), WD repeat containing antisense to TP53 (WRAP53), peroxisomal biogenesis factor 5 (PEX5), RAC-α serine/threonine-protein kinase (AKT1), DDB1 and CUL4 associated factor 15 (DCAF15), SERPINE1 MRNA binding protein 1 (SERBP1), and CBFA2/RUNX1 partner transcriptional corepressor 2 (CBFA2T2) were the most underexpressed genes with significant *p* -values across the two microarray datasets. Fig. 2C illustrates the Venn diagram comparing DEGs sets identified by the individual studies and by meta-analysis. The results obtained by the meta-analysis (1,878 DEGs) are compared with DEGs identified by individual study of ARDS and VTE. Out of the total of 1,878 DEGs identified in our meta-analysis, a total of 390 genes were found to be shared in these two datasets while 1,481 genes from the meta-analysis were shared by VTE dataset individually. A Bayesian meta-analysis model was deployed that combined the probabilities across studies and accounted for the variability across studies. 23

**Table 1:** Enrichment analysis of the shared DEGs in the meta-analysis according to Kobas3.0

Pathway ID	Enriched pathway	Database	Input no.	Background no.	*p* -Value
R-HSA-449147	Signaling by interleukins	Reactome	92	619	3.51E-30
R-HSA-3700989	Transcriptional regulation by TP53	Reactome	71	359	4.07E-30
R-HSA-109582	Hemostasis	Reactome	78	617	7.22E-22
R-HSA-1257604	PIP3 activates AKT signaling	Reactome	48	247	1.55E-20
R-HSA-5633007	Regulation of TP53 activity	Reactome	37	159	2.29E-18
R-HSA-913531	Interferon signaling	Reactome	39	194	2.15E-17
R-HSA-449836	Other interleukin signaling	Reactome	43	275	1.57E-15
R-HSA-446652	Interleukin-1 family signaling	Reactome	29	138	9.83E-14
R-HSA-877300	Interferon gamma signaling	Reactome	23	90	1.10E-12
R-HSA-9020702	Interleukin-1 signaling	Reactome	23	101	8.36E-12
R-HSA-76002	Platelet activation, signaling and aggregation	Reactome	34	260	1.08E-10
R-HSA-1169091	Activation of NF-κβ in B cells	Reactome	18	66	1.25E-10
P00031	Inflammation mediated by chemokine and cytokine signaling	PANTHER	29	201	3.24E-10
R-HSA-1234174	Cellular response to hypoxia	Reactome	18	74	6.08E-10
P00036	Interleukin signaling	PANTHER	18	80	1.78E-09
R-HSA-1234176	Oxygen-dependent proline hydroxylation of hypoxia-inducible factor alpha	Reactome	16	65	4.67E-09
hsa04621	NOD-like receptor signaling	KEGG	25	178	8.51E-09
hsa04657	Interleukin -17 signaling	KEGG	18	93	1.39E-08
R-HSA-9020591	Interleukin-12 signaling	Reactome	13	47	4.02E-08
R-HSA-447115	Interleukin-12 family signaling	Reactome	14	57	4.36E-08
P00047	PDGF signaling	PANTHER	19	124	1.51E-07
R-HSA-6785807	Interleukin-4 and Interleukin-13 signaling	Reactome	17	108	4.80E-07
R-HSA-8950505	Gene and protein expression by JAK-STAT signaling after interleukin-12 stimulation	Reactome	10	38	2.17E-06
R-HSA-448424	Interleukin-17 signaling	Reactome	11	72	6.26E-05
R-HSA-6783783	Interleukin-10 signaling	Reactome	8	47	0.000324
hsa04610	Complement and coagulation cascades	KEGG	10	79	0.000523
R-HSA-1059683	Interleukin-6 signaling	Reactome	4	11	0.001037
R-HSA-9008059	Interleukin-37 signaling	Reactome	5	21	0.0012
R-HSA-8984722	Interleukin-35 signaling	Reactome	4	12	0.001348
R-HSA-6783589	Interleukin-6 family signaling	Reactome	5	24	0.002002
R-HSA-5660668	CLEC7A/inflammasome	Reactome	3	6	0.002313
R-HSA-8854691	Interleukin-20 family signaling	Reactome	5	25	0.002341
P00011	Blood coagulation	PANTHER	6	38	0.00253
R-HSA-2162123	Synthesis of prostaglandins (PG) and thromboxanes (TX)	Reactome	4	15	0.002662
P00030	Hypoxia response via HIF activation	PANTHER	5	26	0.002719
R-HSA-448706	Interleukin-1 processing	Reactome	3	8	0.004333
R-HSA-451927	Interleukin-2 family signaling	Reactome	6	44	0.004852
R-HSA-9020933	Interleukin-23 signaling	Reactome	3	9	0.005641
R-HSA-512988	Interleukin-3, interleukin-5 and GM-CSF signaling	Reactome	6	47	0.006471
R-HSA-9020956	Interleukin-27 signaling	Reactome	3	11	0.008903
R-HSA-1234158	Regulation of gene expression by hypoxia-inducible factor	Reactome	3	11	0.008903
R-HSA-912526	Interleukin receptor SHC signaling	Reactome	4	26	0.014291
R-HSA-2022377	Metabolism of angiotensinogen to angiotensins	Reactome	3	18	0.027605
R-HSA-1266695	Interleukin-7 signaling	Reactome	4	36	0.037224
hsa04614	Renin-angiotensin system	KEGG	3	23	0.048055

Abbreviations: AKT, α serine/threonine-protein kinase; DEG, differentially expressed genes; GM-CSF, gross motor-cerebrospinal fluid; HIF, hypoxia-inducible factor; KEGG, Kyoto encyclopedia of genes and genomes; NF, nuclear factor; NOD, nucleotide-binding, oligomerization domain; PDGF, platelet-derived growth factor; JAK-STAT, janus kinase-signal transducer and activator of transcription; CLEC7A, C-type lectin domain containing 7A; SHC, Src homology 2 domain; PIP3, phosphatidylinositol (3,4,5)-trisphosphate.

Note: Input number signifies the number of hits from the meta-analysis whereas background number is from each curated gene set library. Pathways in the table were ranked based on the adjusted p-value.

### Identification of Overrepresented Biological Pathways and Gene Ontology Terms Using Gene Set Enrichment Analysis

Overrepresented biological pathways associated with the DEGs were evaluated by the gene set enrichment analysis of the Kobas3.0 tool using the complete list of significant upregulated and downregulated DEGs. The results for the enriched biological pathways from various pathway analysis libraries, like the KEGG, Reactome pathway, and PANTHER, were selected with adjusted *p* -value of < 0.05 ( Table 1) . Our meta-analysis showed signaling by interleukins (R-HSA-449147), transcriptional regulation by TP53 (R-HSA-3700989), platelet activation, signaling and aggregation (R-HSA-76002), inflammation mediated by chemokine and cytokine signaling (P00031), cellular response to hypoxia (R-HSA-1234174), and complement and coagulation cascades (HSA04610) as top enriched pathways with adjusted *p* -value of < 0.05 ( Table 1 ).

### Network-Based Meta-analysis for Key Hub Genes

NetworkAnalyst/INMEX was implemented to generate a PPI network by integrating the InnateDB interactome for the complete list of 1,878 DEGs. However, for better visualizations of PPI network, we selected “zero-order” interaction network with 519 nodes connected with 1,181 edges. Supplementary Table S2 provides the complete list of 519 node genes of the zero-order interaction network. The key hub genes were then extracted based on their topological parameters, viz., degree centrality and betweenness centrality, using Cytoscape through the network analyzer. The values of combined ES and *p* -value in the table is derived from the differential expression analysis using INMEX. The most highly ranked nodes across the two datasets based on the network topology measures were tumor protein 53 (TP53; betweenness centrality = 0.294; degree = 60) and ubiquitin B (UBB; betweenness centrality = 0.202; degree = 39) followed by heat shock protein 90 alpha family class-A member 1 (HSP90AA1; betweenness centrality = 0.193; degree = 31), and AKT1 (betweenness centrality = 0.133; degree = 30). Table 2 gives the list of top 20 hub genes of the analysis based on the topological parameter, that is, degree using Cytoscape. To gain further insights into a functionally grouped network of an enriched biological pathway on the DEGs, we specifically selected our top 20 hub genes for pathway enrichment using the ClueGO, a Cytoscape plug-in. ClueGO facilitates the visualization of pathway interaction in the form of network and have highlighted the pathways, such as signaling of Notch, stabilization of P53, activation of nuclear factor (NF)- κβ, and various cell cycle checkpoints, in our study ( Fig. 2E ).

**Table 2:** Top twenty hub genes prioritized based on the topological parameters, that is, degree using Cytoscape

Entrez ID	Symbol	Degree	Betweenness centrality	Closeness centrality	Combined ES	*p* -Value
7157	TP53	60	0.294336	0.369736	−1.6588	0
7314	UBB	39	0.202782	0.349528	0.4802	0.02506
3320	HSP90AA1	31	0.193582	0.352381	0.7022	0.00034
207	AKT1	30	0.133454	0.342366	−1.847	0.000224
983	CD1	27	0.052607	0.318769	0.65994	0.000892
8850	KAT2B	24	0.0692	0.32134	0.6207	0.001954
10594	PRPF8	23	0.033761	0.255172	−1.5438	0
4088	SMAD3	22	0.075699	0.31318	−1.3522	5.34E-12
998	CDC42	20	0.0827	0.289547	0.73897	0.005455
3184	HNRNPD	20	0.035921	0.27364	−0.82441	0.000317
1457	CSNK2A1	19	0.11239	0.315661	−0.51181	0.015109
1660	DHX9	18	0.087967	0.290685	−0.43831	0.047395
5970	RELA	18	0.080218	0.323144	−1.4588	2.12E-13
9978	RBX1	17	0.019157	0.272202	1.5674	0
5710	PSMD4	16	0.002551	0.266324	0.80414	3.19E-05
5706	PSMC6	16	9.23E-04	0.267286	0.44812	0.041198
5690	PSMB2	16	0.00169	0.267286	−0.48723	0.022315
220988	HNRNPA3	16	0.001619	0.239482	−0.5779	0.004609
6429	SRSF4	16	0.005126	0.239593	−0.82971	1.67E-05
5591	PRKDC	16	0.055064	0.324765	−1.1922	6.3E-10

Abbreviations: AKT1, α serine/threonine-protein kinase 1; CD1, cyclin-dependent 1; DHX9, DExH-box helicase 9; ES, effect size; HSP90AA1, heat shock protein 90 alpha family class-A member 1; KAT2B, lysine acetyltransferase 2B; PSMB2, proteasome 20S subunit beta; PSMD4, proteasome 26S subunit, non-ATPase 4; RBX1, RING-box protein 1; RELA, REL-associated protein; TP53, total protein 53; UBB, ubiquitin B; PRPF8, pre-mRNA-processing-splicing factor 8; CDC42, cell division control protein 42 homolog; HNRNPD, heterogeneous nuclear ribonucleoprotein D; CSNK2A1, casein kinase II subunit alpha; PSMC6, proteasome 26S subunit, ATPase 6; HNRNPA3, heterogeneous nuclear ribonucleoprotein A3; SRSF4, serine and arginine rich splicing factor 4; PRKDC, protein kinase, DNA-activated, catalytic subunit.

Note: Expression level (combined ES) and p-value was added from the meta-analysis in the table. The highlighted ones are the genes known to participate in host-viral interactions.

## Discussion

Thromboinflammation, a term that describes inflammation-triggered platelets activation accompanied by endothelium damage, is the one that explains the widespread observation of fibrin deposition and thrombus formation as the consequence of an infection in the poor COVID-19 patient outcome. 11 The higher incidence of ARDS and VTE are found to overlap in the severe COVID-19 patients. 1 2 3 4 To initiate our understanding of the pathophysiological mechanism of this disease and SARS-CoV-2 pathogenicity, we attempted to identify the shared transcriptomic signatures between the two diseases, ARDS and VTE, using gene expression meta-analysis of the two publically available microarray datasets. The gene expression profile dataset for ARDS was derived from the polymorphonuclear leukocytes, 21 while for VTE, the gene expression profile dataset was derived from the whole blood sample. 22 A total of 1,878 DEGs including 736 overexpressed and 1,142 underexpressed genes were identified under the significance threshold of adjusted *p* < 0.05. Supplementary Table S1 presents the complete list of overexpressed and underexpressed DEGs sorted on the basis of combined ES. To elucidate the role of DEGs obtained from the meta-analysis, we performed gene set enrichment analysis and pathway analysis using the comprehensive enrichment library of Kobas3.0. Interestingly, the most enriched pathways among the shared DEGs of the meta-analysis were “signaling by interleukins,” “platelet activation,” “signaling and aggregation,” “inflammation mediated by chemokine and cytokine signaling,” “cellular response to hypoxia,” and “complement and coagulation cascades.” Intriguingly, “inflammasome pathways” and “oxygen-dependent proline hydroxylation of hypoxia-inducible factor α (HIF-1α)” were also present among the significantly enriched pathways in the two datasets. Some of the genes comprising these two pathways such as REL-associated protein (RELA) and caspase 8 of “inflammasome pathways” were among the significantly under expressed genes of our meta-analysis. While phosphatidylinositol-4,5-bisphosphate 3-kinase catalytic subunit β (PIK3CB), phosphoinositide-3-kinase regulatory subunit 1 (PIK3R1), proteasome 26S subunit, non-ATPase 9 (PSMD9), and UBB of “cellular response to hypoxia” pathway were among the significant overexpressed genes of our meta-analysis. Our previous reports have revealed thrombosis due to hypoxia is centrally regulated by a complex network of coagulatory and inflammatory processes, critically linked through HIF-1α. 24 Conceivably, COVID-19 pathogenesis also witnesses inflammation–coagulation–hypoxemia convolutions. SARS-CoV-2 pathology is associated with inflammation-mediated coagulation with the consistent observation of COVID-19 associated coagulopathy as a result of profound inflammatory responses. 2 3 Nevertheless, the procoagulant effect of the virus itself cannot be overlooked and needs thorough investigation. Although a detailed SARS-CoV-2 pathological mechanism is yet to be investigated but the lessons learnt from other virus infections particularly the SARS-CoV-2 reports abnormalities in the fibrinolytic and extrinsic coagulation system. 25 The key intrinsic ways of viruses influencing the coagulation system could be through severe endothelial imbalances promoting prothrombotic phenotypes. Furthermore, the inflammasome-activation feature of SARS-CoV-2 should be considered closely in evaluating intrinsic procoagulant capabilities of SARS-CoV-2 as well. The earlier SARS virus has shown the robust mechanism of NLRP3 inflammasome activation in macrophage by providing a potent signal for its activation. 26 There study identified several other mechanisms, such as induction of endoplasmic reticulum stress, lysosomal damage, and subsequent activation, of the master regulator of the autophagy and lysosome machinery, transcription factor EB by which a SARS-CoV-2 open reading frame activates intracellular stress pathways and targets the innate immune response. Moreover, the series of changes induced by the virus in their host cells could be perceived as endogenous damage-associated molecular patterns (DAMP) to be recognized by NLRP3 inflammasome as well. Hypoxemia, one of the most important cellular changes in the context of COVID-19, is one of the potent DAMP signals for NLRP3 activation. 21 Thus, the inflammation, coagulation, and hypoxemia, as evident in our pathway enrichment analysis, in a highly interrelated fashion could be postulated in enhancing thrombotic condition and disease severity in COVID-19 patients.

Until now, transmembrane angiotensin-converting enzyme 2 (ACE2) through which COVID-19 virus gain entry into the cells, similar to the SARS-CoV-2, has been the focal point for researchers. 27 Our analysis indicated several other nodal points of the host–virus interactions that should be considered evenly. Supplementary Table S2 provides the complete list of 519 nodes genes of the zero-order network as obtained from Cytoscape using network analyzer. At least 9 genes among the top 20 highly ranked hub genes (overexpressed and underexpressed) are known to be involved in host–virus interactions. These were TP53, cyclin-dependent kinase 1 (CDK1), lysine acetyltransferase 2B (KAT2B), SMAD3, DExH-box helicase 9 (DHX9), RELA, RING-box protein 1 (RBX1), and proteasome 20S subunit beta-2 (PSMB2; Fig. 2D ). TP53, the top hub gene of our meta-analysis, is a known target of several viral oncoproteins including SARS-CoV-2 that functionally inactivates it. 28 Their study observed that the human coronaviruses antagonize the viral inhibitor p53 via stabilizing CHY zinc-finger domain-containing 1 (RCHY1) as an interacting partner of the viral SARS unique domain and promoting RCHY1-mediated p53 degradation. Possibly, its downregulation could be utilized by the virus for its own aid in replication and pharmacological rescue of p53 functions might be explored in keeping a check on the virus. 29 Intriguingly, proteasome activator complex subunit 3 (PSME3), a negative regulator of TP53 30 and WRAP53 that gives rise to p53 antisense transcripts and regulates p53 mRNA were among the top downregulated DEGs of the meta-analysis. 31 Subsequently, the pathways related to TP53, such as transcriptional regulation by TP53 and regulation of TP53 activity, were also among the top-enriched pathways ( Table 1 ). All of these parallel results are suggestive of the potential of the TP53 gene function in the understanding of SARS-CoV-2 mechanisms. Similarly, the other top hub genes, such as SMAD3 of transforming growth factor-β (TGF-β) signaling pathway should be investigated for its effects on a large number of biological processes. 32

**Fig. 3:**
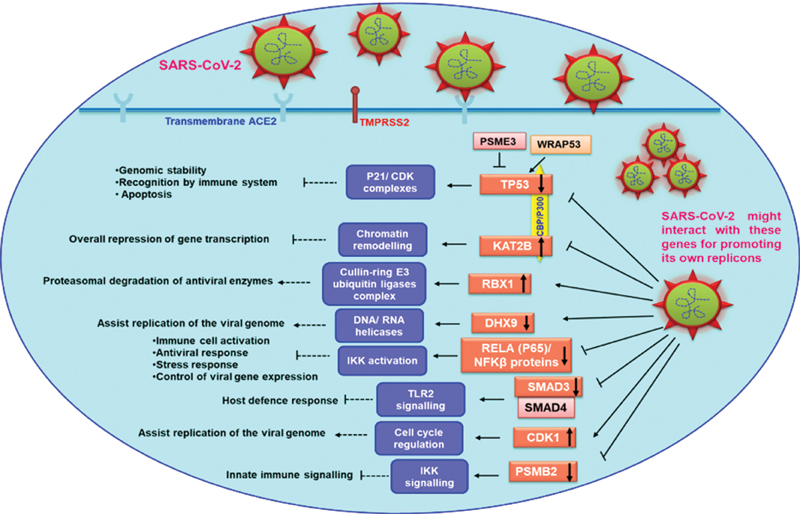
Diagrammatic illustration of host-viral interactions of some of the hub genes that came out from our network-based meta-analysis. The host–virus interactions participation of some of our hub genes is suggestive that these pathological conditions strengthens a favorable environment for virus and further aids in aggravating its viral load and deterioration of patients. ACE2, angiotensin-converting enzyme 2; CDK1, cyclin dependent kinase 1; DHX9, DExH-box helicase 9; IKK, i-κ-kinase; KAT2B, lysine acetyltransferase 2B; NF-κβ, nuclear factor kappa B; P21/CDK, P21/cyclin-dependent kinase; PSMB2, proteasome β subunits 2 family; PSME3, proteasome activator complex subunit 3; RBX1, RING-box protein 1; RELA, REL-associated protein; SARS-CoV-2, severe acute respiratory syndrome-coronavirus-2; TLR2, toll-like receptor 2; TMPRSS2, transmembrane serine protease 2; TP53, tumor protein 53; WRAP53, WD repeat containing antisense to TP53.

RELA (P65) of the NF-κβ family could be manipulated by the virus for modulating NF-κβ signaling that has a profound role in antiviral and antimicrobial responses, immune cell activation, and control of viral gene expression. 33 Cyclin-dependent kinase 1 (CDK1), a key player in cell cycle regulation, is shown to interact with the viral proteins, so that the virus can manipulate the cell cycle for the advantage of their own replication. 34 Likewise, PSMB2, a member of the proteasome β subunits (PSMB) family, has been observed as a negative regulator of the innate immune responses during viral infection through inhibition of RIG-I- and toll-like receptor 3 (TLR3)-mediated type-I interferon responses. 35 RBX1 is an important functional member of cullin-ring E3 ubiquitin ligases complex. 36 37 This complex is observed to be hijacked by the viruses for proteasomal degradation of antiviral enzymes produced as a result of host response against viruses. 36 37 Likewise, DHX9 has been identified as a binding partner of various DNA and RNA viruses to assist replication of the viral genome. 38 DHX9 participates in many cellular pathways including transcription and translation and may enhance virus production or demonstrate the antiviral functions depending on the various stages of the viral life cycle. 38 Similarly, the transcriptional coactivators p300/CBP interacts with an essential viral oncoprotein to suppress the transcription of several genes associated with KAT2B and p53 activation. 39 Perhaps, the host–virus interactions participation of these hub genes in a datasets of ARDS and thrombosis is suggestive that these pathological conditions strengthen a favorable environment for virus and further aids in aggravating its viral load and deterioration of patients ( Fig. 3 ). Our analysis gives insights into these genes and paves the way to understand the various strategies deployed by the virus to improve its replication and spreading.
