# Supplementary material for: Host–Viral Interactions Revealed among Shared Transcriptomics Signatures of ARDS and Thrombosis: A Clue into COVID-19 Pathogenesis
Source: TH Open. 2020 Dec 17;4(4):e403–12. doi: 10.1055/s-0040-1721706 (PMC7746517; doi:10.1055/s-0040-1721706)
Supplement: Supplementary file 1 — Supplementary Material [file 10-1055-s-0040-1721706-s200058.pdf]

Supplementary Table S1 The complete list of DEGs with combined ES and *p*-values

Overexpressed genes			Underexpressed genes		
Name	Combined ES	*p*-Value	Name	Combined ES	*p*-Value
BATF	1.9152	0.016655	*NUP93*	−2.3158	0.01051
ATP5MPL	1.8755	0.0057178	*CASS4*	−2.0853	0.019688
PPA2	1.787	0.017309	*NLRP1*	−2.0309	0.042205
PNP	1.7226	5.51E-05	*WBP11*	−2.0301	8.30E-05
GSTO1	1.6925	0	*WRAP53*	−2.0266	0.049722
PSMA6	1.6761	5.18E-05	*PEX5*	−1.8499	0
TNPO1	1.6642	0.0060162	*AKT1*	−1.847	0.00022464
IGFBP7	1.6631	0.00031067	*DCAF15*	−1.8334	0
NDUFC2	1.6547	0	*SERBP1*	−1.7961	0.049401
MS4A4A	1.61	0.004105	*CBFA2T2*	−1.7949	0
ZDHHC3	1.5938	0.00059391	*ASXL1*	−1.7875	3.28E-05
CD48	1.5877	0	*PPP1R12B*	−1.7352	4.70E-13
RPL27	1.5836	1.61E-07	*TACC1*	−1.725	1.13E-09
RBX1	1.5674	0	*BCOR*	−1.7237	0.020157
PUS3	1.5622	0	*TP53BP1*	−1.7142	0
GTF3A	1.5392	0	*MAP4K2*	−1.7048	4.28E-06
LGALS1	1.5378	8.29E-14	*PTBP1*	−1.7024	1.83E-09
SLC44A1	1.5297	0	*SIN3B*	−1.6951	0
AZI2	1.504	0.034665	*SNCA*	−1.6815	0.048833
HBD	1.5033	0.0036426	*PRKAG2*	−1.6726	0.014448
ESD	1.5016	8.29E-14	*TP53*	−1.6588	0
CRBN	1.4826	8.29E-14	*EVL*	−1.6547	0.03566
LY96	1.4674	1.52E-13	*NOLC1*	−1.639	9.95E-13
RSRC1	1.4612	1.52E-13	*SPATA2*	−1.6324	0.0024947
MYL6B	1.4568	0.010347	*POLM*	−1.628	0
TMEM260	1.4558	2.12E-13	*YLPM1*	−1.6243	0
NANS	1.4508	2.56E-13	*PI4KA*	−1.6228	0
KLHDC10	1.4489	2.56E-13	*PHF8*	−1.6194	2.44E-07
UGCG	1.4471	2.56E-13	*HNRNPA0*	−1.6186	0
UBL5	1.4342	5.72E-05	*UBE4B*	−1.6141	0
DCAF10	1.4322	4.20E-13	*VAMP2*	−1.6098	0
IL6ST	1.4314	0.002628	*ELAVL1*	−1.6071	0
PDCD10	1.4304	4.20E-13	*INPP5B*	−1.6033	0
FCGR1A	1.4265	0.0013478	*RNF19B*	−1.5965	3.06E-12
CCDC69	1.4195	6.33E-13	*SRRT*	−1.5926	0.0034535
FCGR1B	1.4099	8.46E-13	*PSME3*	−1.5757	0
SEC62	1.3925	0.044882	*PSD4*	−1.5749	3.04E-05
SREK1IP1	1.3768	2.47E-12	*GBF1*	−1.5743	0
ARL4A	1.3685	3.19E-12	*TRANK1*	−1.5741	0
ATP6V0E1	1.3675	0.00043622	*DDX21*	−1.5734	0
APOO	1.3549	4.63E-12	*CIRBP*	−1.5636	0.0002942
GABPB1-IT1	1.354	1.39E-09	*SETD1B*	−1.5485	2.82E-12

(*Continued*)

Supplementary Table S1 (*Continued*)

Overexpressed genes			Underexpressed genes		
Name	Combined ES	*p*-Value	Name	Combined ES	*p*-Value
PHACTR2	1.3404	7.30E-12	*TAF4*	−1.5439	0
CKLF	1.3361	8.38E-12	*PRPF8*	−1.5438	0
AHSP	1.3349	1.94E-08	*JAK1*	−1.5369	0.00012552
SP100	1.3322	2.44E-05	*SPG7*	−1.523	0
CTSH	1.3319	0.00055057	*CCNL2*	−1.5164	0
BLOC1S1	1.3307	0.035087	*DEPDC5*	−1.5106	7.37E-11
ATP5MG	1.3304	1.77E-10	*CD4*	−1.5097	0.01466
NDUFB7	1.3301	7.43E-07	*ST6GAL1*	−1.5049	0.00011378
PIK3R1	1.3233	1.29E-11	*ODF2*	−1.5014	8.29E-14
RPL31	1.3187	1.41E-11	*PPIL2*	−1.488	9.32E-08
SELENOF	1.3135	0.0014737	*C2CD3*	−1.4711	1.52E-13
S100A12	1.3093	0.0069196	*SAFB*	−1.471	1.37E-07
COX7A2L	1.3057	2.18E-11	*RELA*	−1.4588	2.12E-13
GYPA	1.2943	1.86E-10	*SAFB2*	−1.4553	2.12E-13
CLIC1	1.2931	3.22E-11	*ZFP36L2*	−1.448	2.56E-13
STX8	1.288	3.71E-11	*CTCF*	−1.448	1.03E-12
CERT1	1.2862	3.91E-11	*BHLHE40*	−1.4408	0.0010802
ZBTB43	1.2822	4.21E-11	*LRRC47*	−1.435	3.76E-13
POMP	1.2768	3.27E-05	*ALOX15*	−1.4318	0.025399
RSL24D1	1.2732	5.73E-11	*ELMO1*	−1.4215	0.026237
ATP5F1E	1.2724	5.73E-11	*ARHGEF18*	−1.4204	1.12E-05
RPL39	1.2694	0.00033825	*PRPF6*	−1.4164	0.001028
RNF7	1.2653	7.12E-11	*PACSIN2*	−1.4101	1.05E-05
DENND1B	1.2643	0.025812	*ABCC1*	−1.3989	6.74E-11
GLRX	1.2562	9.96E-11	*RNPS1*	−1.3984	1.17E-12
TMEM70	1.2501	1.11E-10	*SLC7A6*	−1.3978	1.20E-12
LEPROT	1.2374	1.62E-10	*MAPK8*	−1.3948	0.037874
H4C3	1.2371	0.012622	*RERE*	−1.3929	1.44E-12
MRPL15	1.2359	1.77E-10	*NELFA*	−1.3751	2.62E-12
ANXA1	1.23	1.98E-10	*PHF2*	−1.3702	1.17E-05
C21orf91	1.2279	2.15E-10	*VPS37C*	−1.368	3.19E-12
VAMP5	1.2216	2.53E-10	*RPA1*	−1.3668	0.022494
ENSA	1.2216	2.57E-10	*ANAPC1*	−1.3647	4.98E-05
ANAPC15	1.2139	0.0045486	*MAZ*	−1.3608	4.09E-12
TPT1	1.2123	3.44E-10	*CABIN1*	−1.3579	4.39E-12
MYL12B	1.2117	3.44E-10	*SMAD3*	−1.3522	5.34E-12
LAMTOR5	1.2071	3.94E-10	*RAB11FIP3*	−1.3515	0.0075773
FKBP1B	1.2009	6.17E-05	*HERC1*	−1.3498	2.51E-05
MCTS1	1.1995	0.0023122	*ATP2A3*	−1.3487	0.0017713
AKR1C3	1.197	5.45E-06	*RBM14*	−1.3484	5.68E-12
MRPS31	1.1878	0.042113	*OTUD3*	−1.3481	0.037892
CA1	1.1832	8.23E-10	*PECAM1*	−1.348	5.68E-12

Supplementary Table S1 (*Continued*)

Overexpressed genes			Underexpressed genes		
Name	Combined ES	*p*-Value	Name	Combined ES	*p*-Value
ATP5PD	1.1671	1.36E-09	*IGF2R*	−1.3442	2.56E-07
SUMO4	1.163	0.0018799	*DPF2*	−1.3359	8.38E-12
H2AW	1.1516	2.13E-09	*FAM168B*	−1.3336	8.90E-12
WASHC3	1.139	0.00052709	*ZZEF1*	−1.3334	9.05E-12
COX7A2	1.135	0.036503	*LEF1*	−1.326	3.71E-11
RWDD1	1.1323	1.18E-05	*SPOCK2*	−1.3214	1.41E-11
BLVRB	1.1214	0.012816	*TBP*	−1.3206	1.41E-11
SAR1B	1.1206	5.52E-09	*HP1BP3*	−1.3166	0.045778
LSM6	1.1202	5.65E-09	*AKT2*	−1.3094	0.0091885
UFD1	1.1144	0.023019	*ZHX2*	−1.3089	2.01E-11
TMEM59	1.1133	7.28E-09	*SF3A1*	−1.3026	2.46E-11
DYRK4	1.1098	7.99E-09	*BTG2*	−1.3024	0.0022403
NDUFB1	1.1073	0.0090348	*SNRNP70*	−1.3023	0.0016179
TMA7	1.1071	0.0011521	*EIF4ENIF1*	−1.3018	2.63E-11
CINP	1.107	8.29E-09	*NAB2*	−1.2974	0.018238
GYPB	1.1067	8.30E-09	*MARCKSL1*	−1.2901	3.54E-11
NFAT5	1.1065	0.0021891	*SH2B3*	−1.2899	3.61E-11
RPL41	1.1013	0.028381	*USP19*	−1.2879	3.91E-11
COX6B1	1.1	1.07E-08	*RHOBTB2*	−1.2866	3.91E-11
BCL2A1	1.0988	0.0010304	*MBD1*	−1.2866	0.0021891
PPBP	1.0966	1.13E-08	*YTHDF1*	−1.2854	3.91E-11
POLR1D	1.0948	6.79E-07	*RXRB*	−1.2852	3.91E-11
RPL34	1.0948	0.025788	*CD74*	−1.2821	4.24E-11
ACOT13	1.0946	0.025639	*TMEM94*	−1.2816	4.34E-11
COX6C	1.0925	0.0012791	*MTERF4*	−1.2802	0.031762
ELOC	1.0906	0.0033779	*RIPOR1*	−1.2775	5.02E-11
DNASE1L1	1.0873	0.00031704	*MCM7*	−1.2676	0.015775
OPTN	1.0869	1.52E-08	*SIK3*	−1.2557	9.96E-11
SYT11	1.0848	0.027705	*TJAP1*	−1.2535	1.08E-10
BARD1	1.084	1.64E-08	*COPS7B*	−1.2532	1.04E-10
KPNA3	1.0818	1.80E-08	*IKBKB*	−1.252	1.07E-10
NOL7	1.0792	1.98E-08	*TAOK2*	−1.2517	1.07E-10
ATP5F1C	1.0788	2.04E-08	*ABL1*	−1.2514	1.08E-10
SUMO2	1.0784	0.030994	*SGK1*	−1.2508	4.60E-05
BPGM	1.0725	2.31E-08	*GTF3C2*	−1.2506	1.08E-10
CLIC5	1.0696	2.51E-08	*ZMIZ1*	−1.247	0.00018511
HIGD1A	1.067	0.046142	*NUMA1*	−1.2469	8.23E-05
NLK	1.0638	2.96E-08	*PBXIP1*	−1.2468	2.06E-09
SUCLG1	1.0636	4.59E-06	*DNAJA3*	−1.2453	1.31E-10
CHMP4A	1.0621	3.23E-08	*DPEP2*	−1.2434	1.35E-10
KTN1	1.062	3.15E-08	*PARP1*	−1.2416	1.43E-10
SRI	1.0583	3.51E-08	*BAHD1*	−1.2413	1.41E-08

(*Continued*)

Supplementary Table S1 (*Continued*)

Overexpressed genes			Underexpressed genes		
Name	Combined ES	*p*-Value	Name	Combined ES	*p*-Value
METTL22	1.058	0.0023313	*IL16*	−1.2412	1.55E-10
CAPZA1	1.0579	3.51E-08	*BANP*	−1.2401	8.46E-05
SOD1	1.0579	3.69E-08	*CENPT*	−1.2359	1.07E-06
RPS9	1.0567	3.73E-08	*TRIB2*	−1.2345	1.78E-10
BCAP29	1.0513	4.22E-08	*SLC23A2*	−1.234	1.78E-10
TRNAU1AP	1.0486	4.61E-08	*AKAP17A*	−1.2316	1.92E-10
PIK3CB	1.045	5.16E-08	*PAN2*	−1.2297	2.08E-10
PLGRKT	1.0443	8.34E-06	*TFG*	−1.2294	2.12E-10
RAB29	1.0433	5.24E-08	*UBAP2L*	−1.2284	0.00023705
HBE1	1.0414	5.78E-08	*SDF4*	−1.2276	2.11E-10
RNF11	1.0389	0.015332	*ZNF692*	−1.2233	0.022496
MAIP1	1.0365	0.022568	*NEDD9*	−1.2218	2.53E-10
TNFAIP3	1.029	2.39E-07	*HNRNPU*	−1.2189	0.012529
ACAT1	1.0257	9.13E-08	*PLD3*	−1.2185	2.95E-10
KRAS	1.0177	1.12E-07	*TLE3*	−1.2165	3.00E-10
COPZ1	1.0095	2.76E-06	*KLHL22*	−1.2165	0.00022235
CREG1	1.0094	0.0012051	*RNF4*	−1.2083	3.94E-10
ELL2	1.0064	0.0072417	*PWP2*	−1.2065	0.00027962
ATP5PF	1.0016	1.84E-07	*WWP2*	−1.2055	4.33E-10
UQCRB	1.0011	1.87E-07	*MEF2D*	−1.2053	0.0066041
RPL23AP32	0.99725	2.05E-07	*PILRB*	−1.2045	4.52E-10
MT1X	0.99695	2.05E-07	*RAF1*	−1.2037	4.65E-10
AKR1C2	0.99177	0.045222	*TSPYL2*	−1.2033	0.0062267
SRD5A1	0.99164	2.37E-07	*MAP11*	−1.2029	4.47E-10
PSMD9	0.9907	2.44E-07	*FAM53B*	−1.2007	4.73E-10
GNG11	0.98532	7.21E-05	*PDIA3*	−1.2003	4.76E-10
PYROXD1	0.97766	3.59E-07	*RAB11B*	−1.1989	5.07E-10
NBN	0.97705	3.56E-07	*CALCOCO1*	−1.1988	0.0038023
PJA2	0.97661	3.06E-06	*PBX2*	−1.1986	5.36E-10
C1QB	0.97401	0.0096261	*PDLIM2*	−1.1972	5.38E-10
MINDY2	0.97381	4.05E-07	*MCM3*	−1.1951	5.61E-10
IFI27	0.96935	2.39E-06	*GGA3*	−1.1943	6.00E-10
GPX7	0.9685	0.0049846	*SNUPN*	−1.1941	0.034799
PLEK2	0.96662	4.72E-07	*PRKDC*	−1.1922	6.36E-10
NDUFA8	0.96136	5.70E-07	*TAP2*	−1.1916	6.22E-10
HEBP2	0.96127	5.49E-07	*PHF1*	−1.1872	0.045568
S100A8	0.96089	5.49E-07	*SYMPK*	−1.1871	7.09E-10
CKS1B	0.95972	5.92E-07	*ZNF142*	−1.1859	0.01146
TUBG1	0.95932	5.77E-07	*ZGPAT*	−1.1839	0.0020812
TNFAIP6	0.95789	6.06E-07	*CTDSP2*	−1.1832	0.025399
GTF2E2	0.95478	6.83E-07	*SETX*	−1.1829	0.0020103
XRCC4	0.95046	7.23E-07	*PAK1*	−1.1802	0.034438

Supplementary Table S1 (*Continued*)

Overexpressed genes			Underexpressed genes		
Name	Combined ES	*p*-Value	Name	Combined ES	*p*-Value
UQCRH	0.94925	7.67E-07	*TOM1L2*	−1.1796	9.02E-10
C12orf29	0.94873	7.66E-07	*SH2B2*	−1.1781	0.0010611
EAF2	0.94824	7.77E-07	*RHOT2*	−1.1729	1.13E-09
SLC46A3	0.94667	7.98E-07	*CWC25*	−1.1726	1.14E-09
HTATIP2	0.94636	8.05E-07	*ZXDC*	−1.1714	1.23E-09
TMEM11	0.94312	8.80E-07	*TAF6*	−1.1705	1.22E-09
ATP6V1D	0.94073	9.25E-07	*SKIV2L*	−1.17	4.68E-05
SLPI	0.93948	9.62E-07	*UBE2I*	−1.1682	1.28E-09
TSPAN5	0.93849	9.96E-07	*ITPKB*	−1.1678	1.32E-09
CYP2R1	0.93792	1.02E-06	*DVL2*	−1.1674	1.36E-09
NRIP1	0.93616	0.0027229	*SIRT6*	−1.1652	1.43E-09
SLC16A6	0.9359	1.08E-06	*ZC3H4*	−1.159	5.18E-05
RMDN1	0.93563	1.09E-06	*OSBPL7*	−1.1583	1.73E-09
CDKL1	0.93519	1.07E-06	*FUS*	−1.1572	1.79E-09
DCAF6	0.93505	0.0027032	*SRCAP*	−1.1562	0.028199
HACD1	0.93392	1.11E-06	*AMPD2*	−1.1558	0.020493
ABCG2	0.932	1.17E-06	*ACTN4*	−1.1513	2.13E-09
B3GALT4	0.9303	1.21E-06	*SF3B4*	−1.1498	2.21E-09
MT1H	0.92982	1.24E-06	*C2CD2L*	−1.1495	0.00020433
NSUN3	0.92751	0.0011279	*RPAP1*	−1.1489	2.28E-09
NEDD8	0.92641	1.34E-06	*FBXO46*	−1.1482	7.53E-07
RGCC	0.92522	1.38E-06	*DNMT3A*	−1.1477	2.37E-09
MYL4	0.92477	1.44E-06	*TCF7*	−1.1433	2.80E-09
DAP3	0.92229	0.035452	*PRKAR2B*	−1.1418	0.032289
PERP	0.92132	1.53E-06	*SRRM2*	−1.1417	2.96E-09
ARPC2	0.92102	4.19E-05	*AAMP*	−1.1416	0.0092255
HBQ1	0.92038	0.00055057	*ATG2A*	−1.1409	3.06E-09
TMEM258	0.91842	1.73E-06	*MBOAT7*	−1.1406	1.38E-08
SEC61G	0.91734	1.78E-06	*BRD1*	−1.1399	3.01E-09
NEDD4	0.91717	1.74E-06	*MBTPS1*	−1.1372	3.32E-09
MT1E	0.91574	1.78E-06	*ZMYM3*	−1.1359	3.57E-09
LAMTOR2	0.9156	1.78E-06	*SYNRG*	−1.1325	3.77E-09
KMT5B	0.91489	1.80E-06	*DTX2*	−1.1322	7.23E-07
SNRNP27	0.91058	0.0083262	*LIMD1*	−1.1321	0.02322
USP47	0.91004	2.13E-06	*DBN1*	−1.1307	0.0012155
CADM1	0.90945	2.11E-06	*PARN*	−1.1284	4.31E-09
NDUFAF7	0.90756	2.21E-06	*DYNC1H1*	−1.1255	4.75E-09
ZNF16	0.90746	0.0021508	*ILF3*	−1.1255	0.0023117
SDF2	0.90564	2.33E-06	*LFNG*	−1.1229	5.11E-09
IFT20	0.90521	2.39E-06	*NCAPD3*	−1.1225	5.14E-09
F8	0.9047	2.45E-06	*CLEC16A*	−1.12	0.00066772
GSTZ1	0.90205	2.64E-06	*MZF1*	−1.1199	0.0011156

(*Continued*)

Supplementary Table S1 (*Continued*)

Overexpressed genes			Underexpressed genes		
Name	Combined ES	*p*-Value	Name	Combined ES	*p*-Value
PLA2G12A	0.90087	2.76E-06	*CPSF1*	–1.1171	2.29E-06
LIMS1	0.90013	3.50E-06	*HNRNPUL2*	–1.1161	6.28E-09
TPI1	0.89874	2.84E-06	*NOL9*	–1.1157	6.32E-09
RAP1GDS1	0.89555	3.11E-06	*CD6*	–1.1156	0.0084426
CSTA	0.89462	3.08E-06	*CUL7*	–1.115	0.017324
PCLAF	0.89367	3.21E-06	*HGS*	–1.1095	0.010177
RPA4	0.89347	0.01964	*GGA2*	–1.1084	8.11E-09
MAD2L1BP	0.89211	0.046446	*IKZF5*	–1.1067	8.65E-09
UROD	0.88979	0.00023577	*DDX19A*	–1.1058	9.10E-09
ACAT2	0.88533	0.0056612	*EHMT2*	–1.1042	9.15E-09
SIAH2	0.88423	0.021393	*SMG9*	–1.1034	0.025943
MEOX1	0.88239	0.038948	*PHC1*	–1.0961	1.95E-05
RHAG	0.88063	4.46E-06	*RANBP3*	–1.0904	0.00089254
STAT4	0.87978	4.59E-06	*ACTR1B*	–1.0897	1.48E-08
PRDX2	0.87716	4.89E-06	*100508797*	–1.0889	1.48E-08
EEF1A2	0.87636	4.98E-06	*PACS1*	–1.0879	1.52E-08
OSBPL8	0.87271	5.50E-06	*JARID2*	–1.087	1.51E-08
ENDOD1	0.87233	3.11E-05	*ARFGAP2*	–1.0864	0.010239
PSMA4	0.87204	1.23E-05	*IRF3*	–1.0854	8.89E-08
RTRAF	0.86936	4.60E-05	*TRIO*	–1.0847	1.64E-08
GTF2H5	0.8685	0.0017273	*MOGS*	–1.0823	1.84E-08
ANP32B	0.86794	0.00047905	*JADE2*	–1.0818	0.019723
ACSL6	0.86662	6.45E-06	*SHC1*	–1.0818	0.024837
IL10RB	0.86658	6.54E-06	*PDXDC1*	–1.0778	0.044673
CCRL2	0.86467	6.89E-06	*RBM22*	–1.0775	1.98E-08
IL1A	0.86098	7.85E-06	*DHX30*	–1.075	2.14E-08
ARMCX6	0.85951	0.017234	*QRICH1*	–1.0728	0.00026139
NOP10	0.8588	8.06E-06	*MCCC2*	–1.0727	2.38E-08
DYNLT1	0.85877	8.03E-06	*APBB1*	–1.0718	2.50E-08
JTB	0.85851	0.022783	*PRDM1*	–1.0702	2.60E-08
ATP5PB	0.85793	8.21E-06	*FBXL12*	–1.07	0.020559
PIGK	0.85636	8.47E-06	*LUC7L*	–1.0698	0.0012958
ARPC5L	0.85631	8.47E-06	*RUBCNL*	–1.068	3.97E-07
H2BC3	0.85499	8.76E-06	*SNX19*	–1.0674	2.66E-08
ATP5MC3	0.85287	0.0078139	*PPIF*	–1.0642	2.95E-08
ANG	0.85042	1.02E-05	*PUS7*	–1.0625	0.020397
PPP2R5C	0.85015	1.04E-05	*MAML1*	–1.0621	0.0068847
TFPI	0.84903	1.04E-05	*CHTOP*	–1.0586	3.47E-08
GYPE	0.84853	0.0031916	*RFX1*	–1.0584	3.53E-08
TRAM1	0.8464	1.32E-05	*OAZ2*	–1.0579	3.52E-08
SLC66A2	0.84281	0.0074594	*SSRP1*	–1.0567	3.62E-08
DERA	0.8422	1.22E-05	*PRPF31*	–1.0562	3.64E-08

Supplementary Table S1 (*Continued*)

Overexpressed genes			Underexpressed genes		
Name	Combined ES	*p*-Value	Name	Combined ES	*p*-Value
CSPP1	0.84113	1.28E-05	*MCM3AP*	−1.0548	3.77E-08
DYNC1LI2	0.83993	1.29E-05	*NELFCD*	−1.0539	4.03E-08
CYC1	0.83839	1.33E-05	*SUPT6H*	−1.0522	0.00020749
RPL26	0.83795	0.0080585	*GTPBP3*	−1.0516	0.0098727
TANK	0.83771	0.0041407	*GTF2F1*	−1.0516	0.049037
NDUFA1	0.83695	0.009324	*PRPF19*	−1.0503	4.53E-08
GLA	0.83652	1.40E-05	*KAT5*	−1.0494	4.47E-08
ZNF292	0.83228	1.56E-05	*ARSA*	−1.0494	4.59E-08
HERC4	0.83105	1.61E-05	*RANGAP1*	−1.0492	2.84E-06
HBBP1	0.83095	1.64E-05	*MYCL*	−1.046	4.85E-08
SAT1	0.82987	1.68E-05	*CEP164*	−1.0454	4.95E-08
GSK3B	0.82695	0.02061	*CLK2*	−1.0454	0.00020801
TRIM10	0.82512	1.91E-05	*TRIM62*	−1.0405	6.01E-08
APOBEC3F	0.82481	0.00060239	*AAAS*	−1.0387	6.06E-08
IGF2BP3	0.82467	1.90E-05	*POLRMT*	−1.0379	6.19E-08
ELOVL6	0.82412	1.99E-05	*PDCD11*	−1.0367	6.36E-08
TTC33	0.82392	1.97E-05	*CHD3*	−1.0324	7.36E-08
SCAMP1	0.82368	1.96E-05	*GRAP*	−1.031	4.75E-07
NDUFB3	0.8232	2.00E-05	*PILRA*	−1.0306	7.66E-08
VRK2	0.82154	0.033843	*DNMT1*	−1.0301	0.033548
SNX4	0.82153	0.029178	*FOS*	−1.028	8.58E-08
COQ2	0.82051	5.72E-05	*EDC4*	−1.0278	0.0063305
MRPS28	0.8153	2.40E-05	*KMT2B*	−1.0277	8.71E-08
SRP14	0.81459	2.42E-05	*PIK3R4*	−1.0263	8.92E-08
LAPTM4A	0.812	0.00077955	*CXCR4*	−1.0255	9.06E-08
PAPSS2	0.81187	2.61E-05	*GOLGA3*	−1.0238	9.55E-08
SPATA1	0.80851	0.0023705	*MID1IP1*	−1.0216	1.23E-06
SFT2D2	0.80728	2.90E-05	*POLA1*	−1.0201	0.0014592
MRC1	0.80724	2.90E-05	*TMEM184B*	−1.02	1.06E-07
ATF6	0.80705	2.97E-05	*GTF3C1*	−1.0199	1.08E-07
CCDC51	0.80549	3.03E-05	*PIP4K2B*	−1.0176	1.12E-07
PSMD4	0.80414	3.19E-05	*PI4KB*	−1.0171	1.14E-07
NDUFAF1	0.80374	3.22E-05	*SMARCA4*	−1.0166	0.025639
FECH	0.80307	0.027099	*WIPF1*	−1.01	1.45E-07
PPP1R3C	0.80235	3.26E-05	*TSC1*	−1.0078	1.50E-07
ETHE1	0.80201	0.0013871	*ARF3*	−1.0071	3.61E-07
HOXA10	0.80165	3.30E-05	*EFCAB14*	−1.0056	1.60E-07
HPF1	0.80052	3.47E-05	*MAVS*	−1.0036	1.73E-07
TBC1D8B	0.80012	3.53E-05	*CHMP1B*	−1.0027	1.73E-07
CEBPZ	0.79912	0.015986	*CD5*	−1.0007	1.87E-07
INTS6	0.7977	3.76E-05	*METTL3*	−1.0003	0.0001039
COPS8	0.79646	3.77E-05	*PAXIP1*	−0.99824	1.96E-07

(*Continued*)

Supplementary Table S1 (*Continued*)

Overexpressed genes			Underexpressed genes		
Name	Combined ES	*p*-Value	Name	Combined ES	*p*-Value
GPR63	0.79609	3.81E-05	*NOC2L*	−0.99807	1.97E-07
DYNC1I2	0.79552	3.83E-05	*SLC19A1*	−0.99581	0.013971
VAPB	0.79394	0.00090208	*R3HDM2*	−0.99536	2.16E-07
ABCA1	0.79253	4.20E-05	*CEP72*	−0.99385	3.79E-05
RNASE2	0.7913	0.0078813	*GRK5*	−0.99384	2.27E-07
TMEM106B	0.78959	4.60E-05	*TMEM109*	−0.99369	2.23E-07
TMOD1	0.78645	0.0095987	*ZFAND3*	−0.9931	0.0067903
ATP1B3	0.78492	0.044647	*KLHL3*	−0.9927	2.31E-07
FAM114A2	0.78465	5.14E-05	*USP11*	−0.99161	2.39E-07
ANXA2P3	0.78356	5.18E-05	*NXF1*	−0.99059	2.40E-07
ORMDL2	0.78007	5.71E-05	*SEMA4D*	−0.99008	0.048136
TPMT	0.77796	5.87E-05	*CAPRIN1*	−0.98743	0.0078046
NAPG	0.77572	6.30E-05	*STRN4*	−0.98727	2.72E-07
CAVIN3	0.77413	6.43E-05	*TARBP1*	−0.98696	2.70E-07
CRISP2	0.77397	6.45E-05	*SETD2*	−0.98617	2.72E-07
CHMP5	0.7733	6.54E-05	*NSD2*	−0.98544	2.76E-07
PRDX1	0.77298	0.0028273	*PIM2*	−0.98415	0.025943
SNAP29	0.77084	7.08E-05	*LUC7L2*	−0.98237	3.02E-07
PHLDA3	0.77049	7.05E-05	*ZNF710*	−0.98158	3.20E-07
ATP5ME	0.77012	7.09E-05	*SCAF8*	−0.98142	3.20E-07
TSPO2	0.76902	0.00024212	*ZBTB18*	−0.98085	0.0019429
SPAG7	0.76772	0.0021706	*SIGLEC7*	−0.9785	3.42E-07
COA1	0.76724	0.0049411	*RAB11FIP1*	−0.97833	3.49E-07
CNIH1	0.76689	0.037017	*NCOR2*	−0.97801	3.49E-07
LYRM1	0.76642	7.88E-05	*CNNM4*	−0.97483	0.039306
CTSL	0.7643	8.23E-05	*ZC3H13*	−0.97369	0.0025115
KCTD12	0.76361	8.31E-05	*GEMIN4*	−0.97157	4.23E-07
ZNF654	0.76346	8.42E-05	*RBM5*	−0.97037	4.28E-07
ASAP1-IT1	0.76287	8.42E-05	*PKD1P1*	−0.97013	4.31E-07
PTPN2	0.76259	0.010493	*DDX23*	−0.96994	0.018318
F2R	0.75827	9.40E-05	*MAGED1*	−0.96721	4.72E-07
OXR1	0.75803	9.42E-05	*TELO2*	−0.96687	4.73E-07
23285	0.75802	9.47E-05	*ARHGAP25*	−0.96594	4.77E-07
NDUFV2	0.75587	0.000101	*NCOA1*	−0.96575	4.78E-07
NUAK1	0.7553	0.000101	*CXXC1*	−0.9657	4.97E-07
FHL2	0.75484	0.00010181	*CERS2*	−0.96508	0.0026995
CDC27	0.75473	0.042061	*EMC1*	−0.96472	4.95E-07
CCDC85B	0.75401	0.00010353	*CORO7*	−0.9633	0.0078046
NUCB2	0.75299	0.00010613	*CDK5RAP3*	−0.96202	5.39E-06
AGGF1	0.75284	0.0001066	*URGCP*	−0.961	5.49E-07
ADORA2B	0.74971	0.00011469	*ATP6V1G2*	−0.9597	0.03317
RNF141	0.74958	0.03385	*ABCF3*	−0.95829	5.96E-07

Supplementary Table S1 (*Continued*)

Overexpressed genes			Underexpressed genes		
Name	Combined ES	*p*-Value	Name	Combined ES	*p*-Value
GAL	0.74769	0.0023122	*AKAP8*	−0.95716	0.00014939
MMP1	0.74753	0.00012159	*DTX4*	−0.95703	7.58E-06
ETFB	0.74663	0.00012711	*ATG4B*	−0.95671	6.10E-07
KIF14	0.74382	0.00013393	*PTGER4*	−0.95663	0.0034979
TBCB	0.74346	0.00013393	*C15orf39*	−0.95556	0.047332
PEX2	0.7432	0.00013442	*WDR91*	−0.95357	0.0012417
KRT1	0.74089	0.00014102	*MMS19*	−0.95337	7.06E-07
MKRN1	0.73996	0.00016821	*ZNF532*	−0.95301	4.10E-05
CDC42	0.73897	0.0054548	*IARS1*	−0.95288	7.06E-07
MT2A	0.73886	0.00014795	*TAF15*	−0.95252	0.00043294
ADAP2	0.73696	0.0001547	*SON*	−0.95159	7.07E-07
AP1AR	0.73597	0.00015781	*DARS2*	−0.95132	7.07E-07
ATG3	0.73562	0.036943	*ITPK1*	−0.94999	7.47E-07
SBNO1	0.73506	0.00016504	*SNRK*	−0.94973	7.53E-07
DCLRE1A	0.73344	0.0001685	*PRCC*	−0.94783	7.67E-07
DECR1	0.7319	0.00017569	*MDN1*	−0.94765	4.76E-05
CPA1	0.72828	0.00018924	*RBM42*	−0.94527	8.52E-07
ZNF443	0.72752	0.00019331	*EHMT1*	−0.94505	8.26E-07
APOBEC3G	0.72475	0.00021092	*HMOX1*	−0.94448	8.52E-07
KLF1	0.72228	0.00021867	*ADAM12*	−0.94411	0.00048454
NRAP	0.72199	0.00021981	*PDPR*	−0.94351	0.03984
AVEN	0.71987	0.0026507	*SEPTIN9*	−0.94304	8.78E-07
NRAS	0.71886	0.0023826	*TSC2*	−0.94142	9.16E-07
MGST2	0.71435	0.00026668	*CFAP44*	−0.93877	0.028367
GALNT1	0.71193	0.00027756	*ZBTB48*	−0.93722	0.038901
RAB27B	0.71185	0.00027584	*RBBP4*	−0.93717	1.02E-06
PPP4R4	0.71095	0.00028187	*DIDO1*	−0.93592	1.07E-06
EPM2AIP1	0.71029	0.00028747	*MDC1*	−0.93569	1.08E-06
RHCE	0.7099	0.00028912	*UNC93B1*	−0.93487	1.08E-06
WSB2	0.70816	0.0002989	*ABCG1*	−0.93394	1.11E-06
SRGN	0.70556	0.00031769	*NRBP1*	−0.93382	0.0022462
TRIM36	0.70514	0.00032023	*SENP3*	−0.93299	1.13E-06
GUCY1A1	0.70503	0.00032464	*IRF1*	−0.93284	0.011317
LEPR	0.70464	0.00032292	*PRKD2*	−0.93277	1.14E-06
PCCB	0.70412	0.00032952	*SETD4*	−0.93082	1.20E-06
TRIM2	0.7024	0.010304	*PDIA4*	−0.93047	1.24E-06
HSP90AA1	0.70224	0.00034072	*RCN3*	−0.92885	1.25E-06
AK1	0.70206	0.00034182	*CDV3*	−0.92795	0.00097594
TTC39A	0.70149	0.00034846	*PLPPR2*	−0.92723	1.31E-06
STOML1	0.70032	0.00035608	*TRIM28*	−0.9264	1.35E-06
TRIB3	0.69526	0.00040622	*BRD3OS*	−0.9259	1.35E-06
MT1HL1	0.6942	0.00041836	*APEX2*	−0.9259	1.35E-06

(*Continued*)

Supplementary Table S1 (*Continued*)

Overexpressed genes			Underexpressed genes		
Name	Combined ES	*p*-Value	Name	Combined ES	*p*-Value
SLC35B1	0.69205	0.0076523	*TKFC*	−0.9257	0.023722
ABCD3	0.69195	0.00043428	*CDH2*	−0.92466	1.41E-06
ACOX2	0.69183	0.00043412	*CDK10*	−0.92214	1.50E-06
ARHGEF12	0.69173	0.00043871	*TP73-AS1*	−0.92083	1.56E-06
DPH5	0.68997	0.00045409	*NR1H2*	−0.92075	1.55E-06
RHD	0.68935	0.0004566	*XYLT1*	−0.92002	1.64E-06
HAT1	0.68919	0.0004566	*CD3E*	−0.91929	0.0052006
PKIG	0.68916	0.0004566	*DHX34*	−0.91888	0.017336
LINC00472	0.68847	0.0004638	*TMC6*	−0.91851	1.66E-06
GMFG	0.68839	0.00046468	*TOMM34*	−0.91644	1.78E-06
NUP58	0.68804	0.00047484	*AGO1*	−0.91619	1.79E-06
PLOD2	0.68646	0.00048422	*TUBB*	−0.91583	5.51E-05
DUSP8	0.6863	0.00048407	*AARS1*	−0.91509	1.87E-06
ZMAT3	0.68621	0.00048407	*ZMIZ2*	−0.91276	0.00010805
TARS1	0.68555	0.00095718	*HNRNPAB*	−0.91206	0.045276
ZMYM2	0.68353	0.00051986	*FTSJ1*	−0.90961	2.11E-06
IGFLR1	0.68254	0.00053697	*GTF2H4*	−0.90883	2.19E-06
KRT3	0.67931	0.00056216	*IP6K1*	−0.90769	2.24E-06
MYO6	0.67788	0.00058985	*ZNF592*	−0.90735	2.22E-06
VAMP4	0.67772	0.00058391	*HARS2*	−0.90571	2.38E-06
CENPU	0.67696	0.011445	*PTPN18*	−0.90366	2.48E-06
POLR2J	0.6755	0.011392	*TACC3*	−0.9033	2.53E-06
CDC42BPA	0.67303	0.0038764	*LTK*	−0.90181	0.024746
PTGFR	0.67299	0.00066016	*MIR600HG*	−0.90128	0.0096261
DNAJC6	0.67234	0.00066004	*NPIPA1*	−0.90122	2.61E-06
VSIG4	0.67233	0.019353	*PTP4A3*	−0.90038	2.66E-06
UBE2N	0.67193	0.00066762	*WDR45B*	−0.89991	2.76E-06
CTSE	0.67052	0.00068388	*FCHSD2*	−0.89583	3.02E-06
CDH19	0.66913	0.00070597	*CBFA2T3*	−0.89583	3.02E-06
SERPINB2	0.66686	0.0078435	*SNAPC4*	−0.89518	3.04E-06
KLF5	0.66576	0.00076863	*FAM102A*	−0.89304	3.21E-06
APOBEC3A	0.66519	0.023734	*SNIP1*	−0.8929	3.22E-06
HPRT1	0.66458	0.00079713	*TMEM164*	−0.8926	3.24E-06
FDPS	0.66279	0.00083787	*SPHK2*	−0.89109	3.38E-06
IFT57	0.6623	0.00082829	*MEN1*	−0.89103	3.39E-06
RPL36AL	0.66031	0.00087697	*ZNF207*	−0.88772	3.77E-06
CDK1	0.65994	0.00089154	*ITGA5*	−0.88704	3.80E-06
GPR20	0.6596	0.00087697	*CS*	−0.88656	3.86E-06
H2AC16	0.65917	0.00089287	*TENT4A*	−0.8858	3.91E-06
DNAJA1	0.65812	0.00091273	*ASPSCR1*	−0.88559	3.91E-06
KCNJ2	0.65555	0.00096737	*ZNF280B*	−0.88531	4.07E-06
FNBP1L	0.65539	0.00095691	*METTL17*	−0.88449	4.17E-06

Supplementary Table S1 (*Continued*)

Overexpressed genes			Underexpressed genes		
Name	Combined ES	*p*-Value	Name	Combined ES	*p*-Value
GRAP2	0.65454	0.00097273	*E4F1*	−0.88414	0.0030425
ASCC1	0.65376	0.00099013	*TUBGCP3*	−0.88387	4.27E-06
PBX1	0.65276	0.0010084	*EPHB4*	−0.88383	0.0026199
TMCC2	0.65178	0.0010275	*TFCP2*	−0.8828	0.044875
EIF2AK1	0.65157	0.031287	*RBM15B*	−0.88218	4.38E-06
TBC1D19	0.64975	0.0014593	*DYRK1B*	−0.88074	4.46E-06
VOPP1	0.64872	0.0011068	*HPS4*	−0.87926	4.64E-06
DUSP4	0.64822	0.0011068	*IGHM*	−0.87832	0.031053
DNTTIP2	0.64771	0.0011208	*NUP98*	−0.87775	0.040951
CERNA1	0.64661	0.013424	*100507424*	−0.87681	4.98E-06
CREM	0.64507	0.0011797	*TMEM39B*	−0.87619	0.0098876
CASP5	0.64161	0.001275	*NRCAM*	−0.87544	0.016342
VBP1	0.63837	0.0013615	*XRCC1*	−0.87411	5.46E-06
ABCG5	0.63793	0.0013871	*CAD*	−0.87341	5.46E-06
HDGFL3	0.63681	0.0013984	*LPIN2*	−0.87232	5.59E-06
SH2D1A	0.63619	0.0014427	*ZNF747*	−0.86994	5.97E-06
CTNNAL1	0.63615	0.0014404	*DHX16*	−0.86988	5.94E-06
DCTPP1	0.63565	0.0014474	*KLHL21*	−0.86963	6.06E-06
MPG	0.63434	0.035337	*ADA2*	−0.86929	6.01E-06
SMIM8	0.63234	0.0015282	*CYP4F3*	−0.86928	6.14E-06
TCF4	0.63061	0.0015864	*MED15*	−0.86695	0.010593
SDC4	0.62773	0.0017018	*ATF7IP2*	−0.86664	0.027328
MT1M	0.62504	0.0017871	*AXIN1*	−0.86133	7.58E-06
GTSE1	0.62376	0.0018458	*TYK2*	−0.86106	7.58E-06
SOCS5	0.62363	0.0018387	*LMO7*	−0.85944	0.0034283
TTK	0.62356	0.010416	*GMPPB*	−0.85943	7.90E-06
KAT2B	0.6207	0.0019535	*RALGAPB*	−0.85776	8.22E-06
PLA2G4A	0.61988	0.0019891	*POLR2E*	−0.85476	0.024425
PSMD6	0.61953	0.0020103	*RBM4B*	−0.85431	9.06E-06
SCNM1	0.61862	0.0020465	*NISCH*	−0.85229	0.0072864
FEM1C	0.61814	0.0025298	*DGKQ*	−0.84903	1.05E-05
FYB1	0.61766	0.0021035	*TNFAIP2*	−0.84757	0.000919
MTX1	0.61765	0.0020989	*TBC1D9B*	−0.84754	1.07E-05
RNF2	0.61714	0.049163	*HDC*	−0.84754	8.40E-05
ACTR10	0.61656	0.0021406	*UBA7*	−0.84708	0.035764
ADSL	0.61626	0.047345	*RABEP2*	−0.8466	1.15E-05
CD226	0.61523	0.0021891	*TNFRSF10C*	−0.84498	1.15E-05
PRMT2	0.61425	0.0022403	*PCSK7*	−0.84498	1.17E-05
CENPM	0.61257	0.0023117	*387535*	−0.84434	0.024595
POLR2L	0.61211	0.0023313	*FAM30A*	−0.84416	1.17E-05
SLC6A11	0.61042	0.0023955	*CD27*	−0.84413	1.17E-05
BICD1	0.60983	0.0024238	*EIF4G1*	−0.84295	1.24E-05

(*Continued*)

Supplementary Table S1 (*Continued*)

Overexpressed genes			Underexpressed genes		
Name	Combined ES	*p*-Value	Name	Combined ES	*p*-Value
CLEC4A	0.60912	0.0024641	*VPS52*	−0.84292	1.20E-05
MBNL3	0.6082	0.0025115	*JUND*	−0.84285	1.25E-05
CMA1	0.60769	0.0025199	*ATRN*	−0.84247	3.89E-05
SLFN12	0.60612	0.0026094	*SMARCD1*	−0.84239	1.23E-05
KYAT3	0.60598	0.0026094	*100507397*	−0.84183	1.25E-05
ZNF232	0.60505	0.0026452	*TMEM214*	−0.8402	1.30E-05
PIN4	0.60488	0.0026591	*ALDH3A2*	−0.83979	1.30E-05
S100A9	0.60357	0.0027229	*TAGLN2*	−0.83939	2.52E-05
ANXA2P2	0.60291	0.0027513	*HYOU1*	−0.83859	1.34E-05
NDUFS4	0.60179	0.0028134	*FCGRT*	−0.83853	1.33E-05
NPDC1	0.60114	0.029138	*HMCES*	−0.83807	1.38E-05
PCSK1N	0.6003	0.0029321	*ANKHD1*	−0.8364	1.43E-05
SNX16	0.59971	0.0029992	*IL12RB1*	−0.83477	1.47E-05
RAP1GAP	0.59765	0.0031086	*SLC7A8*	−0.83445	1.49E-05
HOOK1	0.59699	0.0084078	*INTS3*	−0.83351	0.015728
ABCC4	0.59683	0.02191	*FNBP4*	−0.8329	1.55E-05
RBBP8	0.59674	0.035423	*ABCF1*	−0.83146	2.37E-05
HS2ST1	0.59566	0.0032425	*ATP6AP2*	−0.82981	1.67E-05
SPATS2	0.59555	0.030477	*SRSF4*	−0.82971	1.67E-05
NUSAP1	0.59454	0.0033023	*TIAM1*	−0.82874	1.71E-05
GGCT	0.59362	0.0033779	*CXCR1*	−0.82835	0.00012757
C1GALT1	0.59361	0.0033474	*MYO15B*	−0.82767	0.00098269
CAMSAP2	0.5934	0.0033769	*ZNF574*	−0.82765	0.00038247
RFX3	0.59172	0.0034545	*NLGN3*	−0.82762	1.83E-05
ABCA12	0.59015	0.0035638	*GAL3ST4*	−0.82569	1.86E-05
SAP30L	0.58908	0.0036569	*HNRNPD*	−0.82441	0.00031704
PDZK1IP1	0.5888	0.0037108	*TIMM50*	−0.8243	0.00020635
RAB38	0.58831	0.0037137	*GATA2*	−0.82349	1.96E-05
IAPP	0.58708	0.0037956	*PTAFR*	−0.82301	2.02E-05
CAV2	0.58696	0.0037982	*CAPN10*	−0.82279	2.05E-05
SOX1	0.58622	0.0038817	*HLA-F*	−0.8226	2.02E-05
BNIP3	0.58619	0.003889	*GPRASP1*	−0.82259	1.99E-05
100127972	0.58551	0.003938	*TBL3*	−0.82258	2.01E-05
TMEM62	0.58519	0.0039609	*KCTD15*	−0.82128	2.05E-05
HLF	0.58398	0.0040515	*GPI*	−0.82115	2.05E-05
CETN3	0.58363	0.0041002	*MLF2*	−0.82096	2.07E-05
AKT3	0.58252	0.0041851	*PRKCH*	−0.82055	2.07E-05
GATA6	0.581	0.0042788	*DGKZ*	−0.82026	2.11E-05
NRIP3	0.57749	0.004622	*CYP2D6*	−0.82008	2.10E-05
KAT6A	0.57597	0.0047292	*IZUMO4*	−0.81999	2.16E-05
ICAM4	0.57532	0.0048377	*CLINT1*	−0.81855	0.0065978
UCHL5	0.57507	0.0048178	*NFATC1*	−0.81791	2.26E-05

Supplementary Table S1 (*Continued*)

Overexpressed genes			Underexpressed genes		
Name	Combined ES	*p*-Value	Name	Combined ES	*p*-Value
PSMA1	0.57491	0.0048252	*NFYA*	−0.81722	2.27E-05
GPR87	0.57349	0.0049688	*DAZAP1*	−0.81602	2.39E-05
MGC4859	0.573	0.0050069	*KIF13B*	−0.81432	2.46E-05
TMEM87A	0.57246	0.005072	*EGR3*	−0.81323	5.74E-05
TCN1	0.57103	0.0052495	*CRY2*	−0.81292	0.0049588
FRMD4A	0.57054	0.0052634	*BTN2A1*	−0.8127	0.047239
ST8SIA1	0.5685	0.0055052	*INTS9*	−0.81261	2.57E-05
DENND1A	0.56804	0.005542	*EDEM1*	−0.81181	2.63E-05
TRIM66	0.56772	0.0055904	*CYTH2*	−0.81164	2.62E-05
C1orf216	0.56656	0.005663	*CC2D1A*	−0.81048	2.72E-05
CLCN5	0.56561	0.0058231	*LARS2*	−0.81	0.035413
NPEPPS	0.56395	0.0059909	*DCPS*	−0.80969	2.77E-05
LMAN2	0.56394	0.0059742	*ACO2*	−0.80678	0.0072417
PUM3	0.56388	0.0079765	*WDR6*	−0.80522	0.020066
TNNI3K	0.56296	0.0060937	*FCGBP*	−0.8047	3.09E-05
RAB2A	0.56199	0.0061629	*PLCB2*	−0.80358	3.19E-05
RIDA	0.56183	0.0062	*KLHDC3*	−0.80339	3.19E-05
TSPAN7	0.56173	0.0062614	*CRTC1*	−0.80304	3.28E-05
ASGR1	0.5613	0.0062267	*ANKLE2*	−0.80282	3.23E-05
FAM205BP	0.56022	0.0063475	*PITPNM1*	−0.80265	3.25E-05
GSTM3	0.55969	0.0065271	*AACS*	−0.8012	0.01442
CDC34	0.55945	0.0065143	*VWF*	−0.80002	3.46E-05
GOLPH3L	0.55812	0.0065982	*ABTB2*	−0.79884	3.63E-05
LTBP2	0.55717	0.0067386	*DHRS11*	−0.79868	3.63E-05
NCAPG	0.55687	0.0068597	*ZNF76*	−0.7965	3.81E-05
CALCB	0.55558	0.00693	*POLR3E*	−0.79521	3.89E-05
ENC1	0.55505	0.028781	*MFSD12*	−0.79487	0.0068459
NIPSNAP2	0.55466	0.0071425	*MYO9A*	−0.79474	3.95E-05
TNIP3	0.55381	0.0071809	*DDX11*	−0.79454	3.92E-05
NACA2	0.55376	0.015231	*SNN*	−0.79328	0.045568
CDR2L	0.55361	0.0072204	*TRPV1*	−0.7919	4.29E-05
CMAS	0.55279	0.007283	*PPM1G*	−0.78833	4.63E-05
RNF170	0.55266	0.0073317	*CORO1C*	−0.78738	0.016279
ME1	0.55188	0.0074569	*MAN2C1*	−0.78529	4.97E-05
ACVR2B	0.55137	0.0074576	*NOL6*	−0.78481	5.11E-05
KANK2	0.55135	0.007526	*CILP*	−0.78473	5.11E-05
KLK15	0.55109	0.0083	*INTS5*	−0.78341	5.17E-05
MROH9	0.55047	0.007602	*DOCK5*	−0.78329	0.0093801
CBR1	0.54861	0.007837	*AP1B1*	−0.78311	5.34E-05
LHFPL2	0.54773	0.007951	*PLEKHM2*	−0.78261	5.34E-05
HMMR	0.54667	0.0081054	*ADNP*	−0.78153	0.00013393
TRIP4	0.5465	0.0081475	*PCNT*	−0.78148	5.40E-05

(*Continued*)

Supplementary Table S1 (*Continued*)

Overexpressed genes			Underexpressed genes		
Name	Combined ES	*p*-Value	Name	Combined ES	*p*-Value
FGF22	0.54526	0.0083133	*CYHR1*	−0.78119	5.60E-05
PRSS50	0.54499	0.0084142	*PABPN1*	−0.78057	5.52E-05
CPM	0.54476	0.0084426	*TGFBI*	−0.77986	5.67E-05
PF4V1	0.54379	0.0085342	*YIPF3*	−0.77888	5.74E-05
TMX1	0.54269	0.008685	*NUP205*	−0.77798	5.88E-05
TRIM46	0.54023	0.0091979	*PTPN9*	−0.77756	0.044702
MYCN	0.54006	0.0091932	*FUZ*	−0.77712	5.98E-05
PEX3	0.53993	0.0092023	*ABHD4*	−0.7745	6.49E-05
RALGDS	0.53988	0.0091885	*ATP6V1B2*	−0.77413	6.57E-05
H4C13	0.53977	0.0091932	*ELK4*	−0.7741	6.43E-05
PCDHB12	0.53942	0.0092255	*MFAP1*	−0.77392	6.45E-05
ZNF267	0.53927	0.0092758	*AATF*	−0.77343	6.57E-05
SPCS3	0.53906	0.0093124	*TUBGCP2*	−0.77168	0.035928
SAC3D1	0.53863	0.0093816	*SYNJ1*	−0.76943	0.00025436
TMC5	0.53796	0.0094786	*IREB2*	−0.76876	7.36E-05
SNX2	0.53736	0.0096261	*399491*	−0.76862	7.33E-05
CUTC	0.53678	0.0096546	*PPARD*	−0.76847	7.50E-05
AGPAT4	0.536	0.0098595	*LDLRAP1*	−0.76831	7.58E-05
SYCP2	0.53569	0.0098595	*BRD9*	−0.76711	7.59E-05
MRPL18	0.53361	0.013844	*FAM193B*	−0.76656	0.043164
POLR3G	0.53304	0.010411	*TRIM68*	−0.76627	0.0086828
HBB	0.533	0.013181	*FBXO41*	−0.76457	8.15E-05
OAZ3	0.53279	0.010412	*FTO*	−0.76363	8.31E-05
MOCOS	0.53254	0.010576	*FANCE*	−0.76331	8.31E-05
MAN1A2	0.5322	0.012721	*GSTA1*	−0.76302	8.44E-05
RWDD3	0.53156	0.010579	*THBD*	−0.7624	8.66E-05
SCP2	0.53114	0.010716	*SRM*	−0.75964	9.07E-05
ATG4A	0.53108	0.01073	*GAA*	−0.75881	9.40E-05
PALB2	0.53064	0.010732	*RNF40*	−0.75843	9.41E-05
PXMP2	0.53041	0.010775	*FHL3*	−0.75842	0.047843
PARD6B	0.52942	0.01097	*PISD*	−0.75827	9.42E-05
ZNF365	0.52848	0.011199	*SCARB1*	−0.75788	0.011035
TAOK3	0.52841	0.011185	*WWC3*	−0.75739	9.57E-05
LAMTOR3	0.52814	0.011268	*FZD9*	−0.75733	9.59E-05
HBS1L	0.52776	0.011392	*FAM160B2*	−0.75506	0.00010181
RDX	0.5271	0.01144	*BACH2*	−0.7537	0.008215
C9orf40	0.52427	0.012031	*CANT1*	−0.75187	0.0001098
DUSP22	0.52386	0.044	*PLXDC1*	−0.75176	0.00010974
BTG3	0.52283	0.012368	*RBM10*	−0.74939	0.0084426
SLK	0.52192	0.012562	*GCN1*	−0.74863	0.0001213
SCO2	0.52108	0.012897	*KDSR*	−0.74861	0.00011808
INHBC	0.52038	0.012942	*DGAT1*	−0.74736	0.00012159

Supplementary Table S1 (*Continued*)

Overexpressed genes			Underexpressed genes		
Name	Combined ES	*p*-Value	Name	Combined ES	*p*-Value
HELLS	0.52025	0.013087	*MADD*	−0.74685	0.0001252
RFK	0.51996	0.013003	*OGA*	−0.74428	0.00013133
FKBP2	0.51853	0.013415	*DHTKD1*	−0.74405	0.00013139
RAG2	0.51829	0.013455	*INPP5E*	−0.7438	0.00013289
ARG2	0.51788	0.013632	*SRPK2*	−0.7436	0.044665
TBL2	0.51762	0.013632	*ST8SIA4*	−0.74324	0.00013387
SRP9	0.51761	0.013723	*CHFR*	−0.74284	0.00013501
SMCO4	0.51758	0.013723	*KHSRP*	−0.74116	0.00014029
BCL2L13	0.51724	0.013723	*CDK9*	−0.74094	0.00014496
ANKRD40	0.51721	0.013813	*PVR*	−0.7407	0.00014454
MRPL12	0.51587	0.013961	*PRPS1*	−0.74001	0.00014416
CLDN16	0.51437	0.01435	*PLA2G6*	−0.73854	0.00014894
SPC25	0.51406	0.014465	*DET1*	−0.73821	0.0025127
GJD2	0.51402	0.014566	*POLE*	−0.73772	0.00015187
PAQR3	0.51331	0.014625	*SEC31A*	−0.73727	0.00015336
100170939	0.51139	0.015149	*SLC6A12*	−0.73697	0.00015494
C4BPA	0.51128	0.015148	*ST6GALNAC2*	−0.73627	0.00015704
NAT8	0.5111	0.015183	*ARHGAP4*	−0.73554	0.00015937
ACKR2	0.51085	0.015231	*PPRC1*	−0.73473	0.00016294
PLAG1	0.50989	0.01548	*FARSA*	−0.73438	0.00016508
RNF14	0.50866	0.015775	*IQCE*	−0.73373	0.00016955
CPB1	0.50864	0.015845	*HLX*	−0.73358	0.00017057
CYP2W1	0.50852	0.015781	*PITPNA*	−0.73325	0.00016901
B3GALT1	0.50841	0.015845	*SNRPB*	−0.73274	0.00017165
FAP	0.50816	0.015845	*ENGASE*	−0.73236	0.00041601
THAP3	0.50802	0.016035	*KLHL26*	−0.72861	0.00018793
SNX24	0.50667	0.016224	*SP140L*	−0.72859	0.0093601
H4C6	0.50531	0.016519	*VRK3*	−0.72829	0.00018924
GINS2	0.50412	0.016873	*TBRG4*	−0.72809	0.00019018
ANXA2	0.5032	0.01719	*IDH3G*	−0.72744	0.00019434
ZFP37	0.50301	0.017198	*MAGEF1*	−0.72686	0.010361
RPL23AP7	0.50288	0.017316	*FAM120A*	−0.72625	0.0013766
SNX7	0.50244	0.017336	*MED17*	−0.72473	0.00020609
CAMP	0.50199	0.017504	*KLF3*	−0.72397	0.00021551
ST6GALNAC4	0.50148	0.017688	*PACS2*	−0.72328	0.00021903
SEC22B	0.50144	0.017619	*EPHX2*	−0.72217	0.00021936
TMEM45A	0.50084	0.017885	*ERCC3*	−0.72193	0.00022002
PEX26	0.49855	0.018686	*MPV17*	−0.72189	0.00021936
DYNC2LI1	0.49827	0.018592	*RNF144A*	−0.72086	0.00022462
MKKS	0.49814	0.018627	*LRRK1*	−0.71995	0.00022857
ZP2	0.49766	0.018892	*ACACB*	−0.71956	0.00023183
RASSF8	0.49608	0.019258	*BTBD18*	−0.719	0.00023458

(*Continued*)

Supplementary Table S1 (*Continued*)

Overexpressed genes			Underexpressed genes		
Name	Combined ES	*p*-Value	Name	Combined ES	*p*-Value
DAAM1	0.49606	0.01927	*GNE*	−0.71774	0.00073414
SEMA5A	0.49596	0.019272	*TTF2*	−0.7176	0.00024591
FGFR3	0.49418	0.019895	*RFXAP*	−0.71731	0.00024591
DIO1	0.49401	0.020078	*ENG*	−0.71675	0.0002517
IL17B	0.49383	0.02	*TMEM222*	−0.71513	0.00025936
KRT81	0.49329	0.020309	*FGFR1OP*	−0.71484	0.025064
CDH20	0.49224	0.020469	*KATNB1*	−0.71415	0.00026231
IPP	0.49099	0.020938	*ATG13*	−0.7141	0.00026408
GMPS	0.49035	0.021394	*NINJ1*	−0.71393	0.00026244
UBE2E1	0.48864	0.022027	*ADGRL1*	−0.71307	0.0073021
TMEM144	0.48699	0.022387	*MED22*	−0.71094	0.00028408
HPS1	0.4868	0.022687	*CELSR1*	−0.71035	0.00028506
GBP2	0.48649	0.022568	*ERF*	−0.71021	0.00028912
100287590	0.48491	0.023247	*100996496*	−0.70999	0.00028912
RNF13	0.48416	0.023628	*TPCN1*	−0.7096	0.00028912
SLC22A3	0.48313	0.023936	*OSGEP*	−0.7076	0.00030805
EFHC2	0.48309	0.042113	*C1QTNF3*	−0.70745	0.00030404
CCNB1	0.48227	0.02421	*TGFBR2*	−0.70602	0.00031755
ANP32E	0.48226	0.024206	*TRAPPC12*	−0.70418	0.029357
ENPP4	0.48206	0.024511	*ILVBL*	−0.70324	0.00033336
REL	0.48116	0.024627	*SZRD1*	−0.70316	0.00033508
H2AC4	0.48082	0.024982	*TBC1D17*	−0.70252	0.00033837
SCFD1	0.48062	0.025064	*POMT1*	−0.70199	0.00034459
UBB	0.48025	0.025064	*SPATA20*	−0.69968	0.00036334
PPM1E	0.47985	0.025324	*EMD*	−0.69856	0.00037105
CASK	0.47972	0.025349	*KCNK7*	−0.69789	0.00037837
MRGBP	0.47808	0.025973	*EIF2S3*	−0.69784	0.00037967
MYOM2	0.47803	0.025943	*MAPK7*	−0.69762	0.0011694
STK17B	0.4751	0.027099	*KIAA1109*	−0.69683	0.00039445
SYCE1L	0.47454	0.027328	*57051*	−0.69672	0.00039129
GPRC5B	0.47418	0.027568	*TTN*	−0.69588	0.00039835
TMEM255A	0.47251	0.028412	*FAM86C1*	−0.69563	0.00039664
GNA12	0.47234	0.028367	*CROCC*	−0.69543	0.011414
VENTXP1	0.47186	0.028794	*CYP3A5*	−0.69403	0.010982
DGUOK	0.47152	0.028629	*RFPL1S*	−0.69372	0.00041455
SPTA1	0.47111	0.028847	*WARS1*	−0.6925	0.000432
AUNIP	0.46998	0.029388	*PTPRO*	−0.69236	0.016224
STS	0.46987	0.029388	*DELE1*	−0.69228	0.00042857
CYP51A1	0.46902	0.029947	*SUPT5H*	−0.69212	0.0027714
NCOA3	0.46785	0.030683	*SUGP1*	−0.69201	0.019934
DESI1	0.46694	0.03079	*BAZ1B*	−0.69179	0.005539
CRB1	0.46684	0.030802	*ADARB1*	−0.69177	0.00043727

Supplementary Table S1 (*Continued*)

Overexpressed genes			Underexpressed genes		
Name	Combined ES	*p*-Value	Name	Combined ES	*p*-Value
TSPAN12	0.46625	0.031084	*KIT*	−0.69175	0.00043412
PRUNE2	0.46592	0.031233	*BAIAP2*	−0.69075	0.037379
FRK	0.46559	0.031718	*CCDC9*	−0.69044	0.016041
KRT8	0.46491	0.031778	*TWNK*	−0.69008	0.029388
S100A10	0.46446	0.031927	*DROSHA*	−0.68995	0.00046018
MUC2	0.46413	0.032196	*CCNJL*	−0.68905	0.00045842
HBEGF	0.46227	0.033213	*CALCOCO2*	−0.68857	0.00046468
BIRC2	0.46221	0.033213	*ELAC2*	−0.68823	0.00046693
ABITRAM	0.46193	0.03336	*CD46*	−0.68811	0.00046693
FAM149A	0.46081	0.03403	*ZNF185*	−0.68792	0.00046736
XPNPEP2	0.4598	0.034441	*NUP210*	−0.68708	0.00047532
H3-4	0.45974	0.03459	*OTUB1*	−0.68686	0.00048407
ALLC	0.4597	0.034438	*TBC1D13*	−0.68647	0.00048407
KRT12	0.45889	0.034896	*SGPL1*	−0.68593	0.00048454
CYP7A1	0.45772	0.035531	*APBA2*	−0.68389	0.00050969
ZBTB7C	0.45677	0.036152	*INPP5A*	−0.68358	0.00052534
PRR16	0.45637	0.036151	*EIF4EBP2*	−0.68346	0.00051986
ASPN	0.45607	0.036759	*HUWE1*	−0.68314	0.00052476
COLEC10	0.45522	0.037017	*VAMP1*	−0.68276	0.00091009
RABIF	0.45505	0.036995	*HERPUD1*	−0.68271	0.00052837
TGM2	0.45414	0.037331	*MX2*	−0.68194	0.00053157
BAG1	0.45362	0.037725	*DRG2*	−0.68138	0.00053734
KLRG1	0.4528	0.038485	*NIPBL*	−0.68138	0.0005394
ADAMTS3	0.45209	0.038635	*NIPAL2*	−0.68127	0.045123
GSTK1	0.45175	0.038991	*CLCF1*	−0.68112	0.00053945
CST1	0.45174	0.038819	*PGGHG*	−0.68044	0.00055129
RANBP17	0.45171	0.038803	*FFAR2*	−0.67925	0.0085086
SLC13A1	0.4507	0.039505	*TXNRD3*	−0.67874	0.00057869
POGLUT2	0.4499	0.039931	*SLC27A3*	−0.67717	0.00075242
9142	0.44972	0.040031	*KCTD20*	−0.67713	0.00059302
ADGRE1	0.44919	0.040595	*VPS13B*	−0.67703	0.00059916
ZNF35	0.44918	0.040691	*IPO4*	−0.67681	0.00059354
SCGN	0.44859	0.040733	*LPXN*	−0.67479	0.00063933
ALDH5A1	0.44835	0.040933	*DDB1*	−0.67381	0.00064692
RABL3	0.44826	0.04102	*HIVEP3*	−0.6718	0.00066605
PSMC6	0.44812	0.041198	*TPM2*	−0.67124	0.00067485
FBXO31	0.44677	0.042211	*SLC5A7*	−0.67118	0.00067862
VCAN	0.44656	0.042113	*STX3*	−0.67073	0.00069289
BIRC3	0.44643	0.042289	*YEATS2*	−0.66908	0.00070899
PSMD1	0.44564	0.042767	*HOXB2*	−0.66762	0.0007385
ERBB4	0.44513	0.042939	*KCNQ1*	−0.66743	0.00074434
C17orf75	0.44483	0.043109	*MAST3*	−0.66636	0.0056925

(*Continued*)

Supplementary Table S1 (*Continued*)

Overexpressed genes			Underexpressed genes		
Name	Combined ES	*p*-Value	Name	Combined ES	*p*-Value
NLGN4Y	0.44461	0.043431	*SIPA1L1*	−0.66556	0.016277
RAB25	0.44446	0.043373	*TSPYL5*	−0.66549	0.0007683
PLAAT1	0.44234	0.044882	*EMSY*	−0.66207	0.00083189
ATP6V1E1	0.44215	0.044857	*DDX10*	−0.66049	0.016903
NMI	0.44204	0.044954	*TCOF1*	−0.66013	0.00086696
EIF5A2	0.44197	0.044931	*FXR2*	−0.65932	0.00088769
RUNDC3A	0.4409	0.045619	*HNRNPDL*	−0.65928	0.00089076
IGF2BP2	0.44053	0.045883	*CCHCR1*	−0.65848	0.00089795
ZNF385D	0.44033	0.045883	*BPTF*	−0.65821	0.00096211
LSM2	0.43988	0.04624	*PPP4C*	−0.65746	0.00091284
FLRT3	0.43942	0.046476	*PLEKHJ1*	−0.6574	0.00091517
RFPL3	0.43846	0.047602	*CCNG2*	−0.65507	0.00097594
SEPTIN10	0.43822	0.047489	*WLS*	−0.65468	0.00097059
WASHC4	0.43798	0.0477	*NCBP3*	−0.65433	0.00097594
RPL38	0.43774	0.047983	*FKRP*	−0.65402	0.00098903
CCL8	0.43735	0.04796	*PIP4K2C*	−0.65308	0.0011605
H3C8	0.4364	0.048516	*C7orf26*	−0.65265	0.0010109
FBXL14	0.43593	0.048927	*CDK20*	−0.65233	0.0010148
NFKBIE	0.43512	0.04981	*TULP4*	−0.65071	0.0010515
NEK2	0.43489	0.04963	*PEX14*	−0.6506	0.0010548
GRIK4	0.4348	0.049877	*UCKL1*	−0.6504	0.0010799
SLC4A4	0.43477	0.049687	*ABHD6*	−0.65019	0.0010613
–	–	–	*KNTC1*	−0.6498	0.0010695
–	–	–	*WDR60*	−0.64836	0.0011208
–	–	–	*PPP6R1*	−0.64783	0.0011208
–	–	–	*RAB5A*	−0.64776	0.0011291
–	–	–	*BCL11B*	−0.64759	0.010972
–	–	–	*LRIG2*	−0.64757	0.042451
–	–	–	*SAMD4B*	−0.64709	0.0011293
–	–	–	*PDE4A*	−0.64663	0.0011429
–	–	–	*EEF2*	−0.64594	0.0011563
–	–	–	*STMN3*	−0.64591	0.019032
–	–	–	*RRP1B*	−0.64585	0.0011605
–	–	–	*VDR*	−0.64541	0.039164
–	–	–	*LINC00342*	−0.64509	0.0011757
–	–	–	*NBEA*	−0.64434	0.0012043
–	–	–	*EDRF1*	−0.64372	0.0012123
–	–	–	*HLA-DOA*	−0.64366	0.001215
–	–	–	*GPA33*	−0.64118	0.0012831
–	–	–	*ZNF589*	−0.64096	0.0012833
–	–	–	*COL5A3*	−0.64086	0.0012992
–	–	–	*PTGS1*	−0.63955	0.0013227

Supplementary Table S1 (*Continued*)

Overexpressed genes			Underexpressed genes		
Name	Combined ES	*p*-Value	Name	Combined ES	*p*-Value
–	–	–	*RGS2*	−0.63952	0.013823
–	–	–	*ARMC6*	−0.6386	0.0013498
–	–	–	*SPOUT1*	−0.63786	0.0013766
–	–	–	*TSPAN4*	−0.63765	0.0013952
–	–	–	*USP34*	−0.6368	0.0014153
–	–	–	*TTLL4*	−0.63672	0.0014053
–	–	–	*LONP1*	−0.63608	0.001437
–	–	–	*SPINK5*	−0.63599	0.047283
–	–	–	*KIAA0100*	−0.63585	0.0014427
–	–	–	*SUPV3L1*	−0.63561	0.0014427
–	–	–	*DENND1C*	−0.63528	0.0014427
–	–	–	*ERI3*	−0.6352	0.0014478
–	–	–	*CASP8*	−0.6345	0.0014592
–	–	–	*ESRRA*	−0.6342	0.0014684
–	–	–	*CXCR5*	−0.63416	0.0014794
–	–	–	*MOAP1*	−0.6329	0.0015311
–	–	–	*TRAPPC9*	−0.63145	0.0015704
–	–	–	*PPP1R13B*	−0.63118	0.0015725
–	–	–	*NACC2*	−0.62905	0.0016784
–	–	–	*LY75*	−0.62854	0.0016718
–	–	–	*CHKB*	−0.62755	0.0017219
–	–	–	*LMAN1*	−0.62662	0.0017304
–	–	–	*TFIP11*	−0.6265	0.0017435
–	–	–	*LOC441601*	−0.62621	0.0017484
–	–	–	*ZNF264*	−0.62587	0.0017649
–	–	–	*TSPAN32*	−0.62504	0.001802
–	–	–	*PSKH1*	−0.62467	0.0018386
–	–	–	*ZFY*	−0.62446	0.0018157
–	–	–	*NELL2*	−0.62239	0.0018898
–	–	–	*ADAT1*	−0.62222	0.0018958
–	–	–	*ARHGEF40*	−0.62193	0.001905
–	–	–	*POLD2*	−0.62181	0.0025421
–	–	–	*RAI1*	−0.62139	0.0019352
–	–	–	*OGFOD2*	−0.62128	0.0019317
–	–	–	*PIP5K1C*	−0.62126	0.0019508
–	–	–	*ARFRP1*	−0.62036	0.0083513
–	–	–	*KIAA0232*	−0.61994	0.043164
–	–	–	*CSNK2B*	−0.61953	0.0020073
–	–	–	*PRB1*	−0.61638	0.0021406
–	–	–	*ELP3*	−0.61561	0.0021859
–	–	–	*TRIM48*	−0.6154	0.0021818
–	–	–	*ACADVL*	−0.61285	0.0022971

(*Continued*)

Supplementary Table S1 (*Continued*)

Overexpressed genes			Underexpressed genes		
Name	Combined ES	*p*-Value	Name	Combined ES	*p*-Value
–	–	–	*HNRNPUL1*	−0.6125	0.0023122
–	–	–	*ICOSLG*	−0.61241	0.0023122
–	–	–	*SPPL2B*	−0.61199	0.0023635
–	–	–	*TESK2*	−0.61199	0.021572
–	–	–	*PNMA3*	−0.61139	0.0023709
–	–	–	*ALDH9A1*	−0.61084	0.0024238
–	–	–	*IMP3*	−0.61006	0.017494
–	–	–	*UBR2*	−0.60948	0.0024704
–	–	–	*HNRNPH1*	−0.60898	0.0024714
–	–	–	*MYO5C*	−0.60858	0.0025105
–	–	–	*CYP2J2*	−0.60846	0.0024947
–	–	–	*RNF185*	−0.6082	0.0025127
–	–	–	*HARS1*	−0.60712	0.0025731
–	–	–	*G3BP2*	−0.60708	0.0025561
–	–	–	*PPP4R1*	−0.60652	0.0026087
–	–	–	*SCARF1*	−0.60626	0.0026199
–	–	–	*LRAT*	−0.60622	0.0026077
–	–	–	*FOXH1*	−0.6058	0.0026101
–	–	–	*SNW1*	−0.60534	0.0026316
–	–	–	*SLC2A10*	−0.60522	0.0026591
–	–	–	*LGALS9*	−0.60461	0.002665
–	–	–	*LIMD2*	−0.6026	0.0028198
–	–	–	*SLC30A6*	−0.60209	0.0027994
–	–	–	*LMO3*	−0.60159	0.002849
–	–	–	*ZNF814*	−0.60132	0.0028478
–	–	–	*PSMD11*	−0.60114	0.0028868
–	–	–	*PPFIA1*	−0.59987	0.0029228
–	–	–	*ZNF214*	−0.59709	0.0031241
–	–	–	*MTMR14*	−0.59628	0.0031771
–	–	–	*CBLL1*	−0.59554	0.0032425
–	–	–	*DFFB*	−0.59455	0.0033023
–	–	–	*CLN6*	−0.59452	0.0032853
–	–	–	*HNRNPH3*	−0.59421	0.0033023
–	–	–	*ZNF205-AS1*	−0.59203	0.0034352
–	–	–	*UBN1*	−0.59061	0.0035396
–	–	–	*CCL19*	−0.59045	0.0035576
–	–	–	*CDK2AP2*	−0.59009	0.0035638
–	–	–	*TRAK1*	−0.58925	0.0036569
–	–	–	*IDH3A*	−0.58841	0.003693
–	–	–	*TARS2*	−0.58579	0.003889
–	–	–	*SARS2*	−0.58557	0.0039346
–	–	–	*SYNDIG1*	−0.58365	0.0040545

Supplementary Table S1 (*Continued*)

Overexpressed genes			Underexpressed genes		
Name	Combined ES	*p*-Value	Name	Combined ES	*p*-Value
–	–	–	*EPHB6*	−0.58285	0.004145
–	–	–	*ZIM2*	−0.5804	0.0043902
–	–	–	*UIMC1*	−0.58	0.0044664
–	–	–	*C1orf159*	−0.57921	0.0044664
–	–	–	*ZW10*	−0.57891	0.0044703
–	–	–	*MRPL4*	−0.57848	0.0045643
–	–	–	*RBM28*	−0.57827	0.0045302
–	–	–	*HNRNPA3*	−0.5779	0.0046098
–	–	–	*NUP50*	−0.57779	0.0046061
–	–	–	*GDF3*	−0.57716	0.0046229
–	–	–	*MGAT3*	−0.57646	0.0046964
–	–	–	*SYNGR2*	−0.57585	0.0047448
–	–	–	*ENTR1*	−0.57527	0.0048252
–	–	–	*TMEM63A*	−0.57253	0.0050545
–	–	–	*PLXNB2*	−0.57191	0.0051274
–	–	–	*GNL3L*	−0.57156	0.0051912
–	–	–	*TOMM22*	−0.57148	0.0052125
–	–	–	*GNAT2*	−0.57113	0.005252
–	–	–	*NCAM2*	−0.57106	0.0052495
–	–	–	*TEX261*	−0.57009	0.0053806
–	–	–	*TRAPPC10*	−0.5694	0.0053859
–	–	–	*TRIM26*	−0.56857	0.005527
–	–	–	*GPS1*	−0.56804	0.0055136
–	–	–	*GALNT8*	−0.5675	0.0056564
–	–	–	*SLC6A16*	−0.56741	0.048627
–	–	–	*ERCC5*	−0.56633	0.0057042
–	–	–	*CAND2*	−0.56605	0.0058231
–	–	–	*JMJD1C*	−0.56575	0.0057433
–	–	–	*LRRN3*	−0.56504	0.0058471
–	–	–	*CERK*	−0.56434	0.005916
–	–	–	*ADAM11*	−0.56418	0.006007
–	–	–	*CD22*	−0.56408	0.0059753
–	–	–	*PDIA6*	−0.5637	0.006007
–	–	–	*WDR46*	−0.56364	0.0060162
–	–	–	*GPATCH3*	−0.56346	0.037017
–	–	–	*SQSTM1*	−0.56311	0.006104
–	–	–	*CIZ1*	−0.56262	0.0061519
–	–	–	*DOP1B*	−0.56233	0.0061906
–	–	–	*DXO*	−0.56213	0.0061629
–	–	–	*TRMT2B*	−0.56168	0.0061906
–	–	–	*SWAP70*	−0.56119	0.0062916
–	–	–	*SGTA*	−0.56053	0.0063172

(*Continued*)

Supplementary Table S1 (*Continued*)

Overexpressed genes			Underexpressed genes		
Name	Combined ES	*p*-Value	Name	Combined ES	*p*-Value
–	–	–	*PELI2*	−0.56047	0.0063329
–	–	–	*GPM6B*	−0.55965	0.0064222
–	–	–	*TXK*	−0.55856	0.0065578
–	–	–	*ZCCHC8*	−0.55704	0.017798
–	–	–	*UBE2M*	−0.55614	0.0068624
–	–	–	*SPIB*	−0.55556	0.0069913
–	–	–	*ADRM1*	−0.55524	0.0069779
–	–	–	*AP5Z1*	−0.55434	0.0071305
–	–	–	*SLC25A11*	−0.55403	0.0072204
–	–	–	*WDR55*	−0.55363	0.0071935
–	–	–	*MSH6*	−0.55339	0.016128
–	–	–	*VAMP3*	−0.55291	0.0073067
–	–	–	*CASC3*	−0.55288	0.0072982
–	–	–	*HTT*	−0.55288	0.016927
–	–	–	*MDH2*	−0.55226	0.0073519
–	–	–	*SLC25A1*	−0.55188	0.0074278
–	–	–	*CYP27A1*	−0.55173	0.0074569
–	–	–	*UCP2*	−0.55054	0.0076079
–	–	–	*LRBA*	−0.55042	0.007602
–	–	–	*ABCC6*	−0.54964	0.0077006
–	–	–	*SLC37A1*	−0.54923	0.0078139
–	–	–	*CACNA1D*	−0.54845	0.021391
–	–	–	*POLA2*	−0.5482	0.007917
–	–	–	*FOCAD*	−0.54668	0.0081095
–	–	–	*VAC14*	−0.54614	0.0082028
–	–	–	*MON1B*	−0.54356	0.0086468
–	–	–	*RPS6KA4*	−0.54313	0.0086516
–	–	–	*YY1*	−0.5429	0.0086686
–	–	–	*PTPN11*	−0.54241	0.0087296
–	–	–	*IRF9*	−0.54159	0.0088838
–	–	–	*SDHAP1*	−0.54156	0.0089266
–	–	–	*HAL*	−0.53896	0.0093149
–	–	–	*DKKL1*	−0.53734	0.0096283
–	–	–	*TEX10*	−0.53704	0.0096283
–	–	–	*BCKDK*	−0.53699	0.0096512
–	–	–	*IRF8*	−0.53582	0.0098595
–	–	–	*GORASP1*	−0.53555	0.010029
–	–	–	*SLC5A6*	−0.5355	0.0098684
–	–	–	*SNTB1*	−0.53275	0.030173
–	–	–	*DENND2B*	−0.53268	0.010468
–	–	–	*ZDHHC18*	−0.53178	0.010553
–	–	–	*CAMK2G*	−0.53152	0.010595

Supplementary Table S1 (*Continued*)

Overexpressed genes			Underexpressed genes		
Name	Combined ES	*p*-Value	Name	Combined ES	*p*-Value
–	–	–	*HEY1*	−0.53128	0.010705
–	–	–	*MYO5A*	−0.52998	0.01091
–	–	–	*CYP4F2*	−0.52975	0.01091
–	–	–	*RAD52*	−0.52975	0.010924
–	–	–	*ABAT*	−0.52773	0.011302
–	–	–	*H3C6*	−0.52761	0.011317
–	–	–	*FAM86B1*	−0.52739	0.011358
–	–	–	*PAPOLB*	−0.52739	0.011489
–	–	–	*FRMD8*	−0.52671	0.011462
–	–	–	*EIF2B1*	−0.52603	0.011755
–	–	–	*ARNTL*	−0.52522	0.04733
–	–	–	*WDR19*	−0.52496	0.011878
–	–	–	*AKAP12*	−0.52463	0.01195
–	–	–	*SEC24B*	−0.5242	0.012086
–	–	–	*CDC25B*	−0.52353	0.012204
–	–	–	*AHCTF1*	−0.52162	0.01264
–	–	–	*UNC119B*	−0.52085	0.012804
–	–	–	*HDLBP*	−0.51904	0.013225
–	–	–	*MDM2*	−0.51876	0.013329
–	–	–	*GABRA4*	−0.51851	0.023276
–	–	–	*SMPDL3B*	−0.51823	0.021627
–	–	–	*TESMIN*	−0.51755	0.013585
–	–	–	*ATAD3A*	−0.51693	0.013776
–	–	–	*AGPAT3*	−0.51683	0.013844
–	–	–	*TUBGCP5*	−0.51663	0.013828
–	–	–	*CPT2*	−0.51637	0.013838
–	–	–	*NSFL1C*	−0.51586	0.013941
–	–	–	*PRSS53*	−0.51537	0.016318
–	–	–	*TREML2*	−0.51485	0.01435
–	–	–	*NFKBIB*	−0.51422	0.014416
–	–	–	*CLUH*	−0.51415	0.014413
–	–	–	*KRTAP4-7*	−0.51321	0.014616
–	–	–	*FOSB*	−0.51313	0.01466
–	–	–	*KRT23*	−0.51291	0.01466
–	–	–	*LRRC59*	−0.51236	0.014796
–	–	–	*CD19*	−0.51222	0.014831
–	–	–	*CSNK2A1*	−0.51181	0.015109
–	–	–	*A1CF*	−0.51172	0.015109
–	–	–	*PML*	−0.51123	0.015122
–	–	–	*CLIP2*	−0.5111	0.015125
–	–	–	*LINC00939*	−0.50992	0.015453
–	–	–	*PPEF1*	−0.50952	0.015637

(*Continued*)

Supplementary Table S1 (*Continued*)

Overexpressed genes			Underexpressed genes		
Name	Combined ES	*p*-Value	Name	Combined ES	*p*-Value
–	–	–	*KLK6*	−0.50939	0.015728
–	–	–	*P2RX5*	−0.50903	0.015804
–	–	–	*PFKM*	−0.50835	0.015818
–	–	–	*ANXA11*	−0.50828	0.015818
–	–	–	*PRKY*	−0.50807	0.015955
–	–	–	*ZNF646*	−0.50797	0.015903
–	–	–	*ZNF638*	−0.50792	0.015887
–	–	–	*BLCAP*	−0.50779	0.015909
–	–	–	*WHRN*	−0.50724	0.016061
–	–	–	*REC8*	−0.50717	0.016235
–	–	–	*MEPCE*	−0.50696	0.016167
–	–	–	*ZNF202*	−0.50675	0.016167
–	–	–	*SLC49A3*	−0.50646	0.016301
–	–	–	*SNRNP40*	−0.50621	0.016318
–	–	–	*STX4*	−0.50526	0.01652
–	–	–	*PVT1*	−0.50489	0.016655
–	–	–	*C6orf62*	−0.50405	0.016999
–	–	–	*IL25*	−0.50283	0.017324
–	–	–	*G3BP1*	−0.5016	0.017577
–	–	–	*MRPL44*	−0.50102	0.017785
–	–	–	*LAMP2*	−0.5008	0.017798
–	–	–	*CST3*	−0.50018	0.018018
–	–	–	*NAA16*	−0.49988	0.018209
–	–	–	*GOT2*	−0.49923	0.018306
–	–	–	*C2CD2*	−0.49909	0.01834
–	–	–	*SIRT3*	−0.49904	0.018347
–	–	–	*SNX17*	−0.49855	0.018507
–	–	–	*MAN2B1*	−0.49836	0.018816
–	–	–	*MAGEA3*	−0.49825	0.01881
–	–	–	*MTCL1*	−0.49739	0.019048
–	–	–	*STARD3*	−0.49712	0.019171
–	–	–	*ESRP1*	−0.49702	0.019098
–	–	–	*APLNR*	−0.49698	0.019171
–	–	–	*RUBCN*	−0.49632	0.019253
–	–	–	*VCP*	−0.4962	0.019416
–	–	–	*MTR*	−0.49618	0.019246
–	–	–	*SOAT1*	−0.49553	0.019476
–	–	–	*POP1*	−0.49536	0.019476
–	–	–	*ADAM29*	−0.4947	0.019717
–	–	–	*KDM3A*	−0.4938	0.020108
–	–	–	*FASTKD5*	−0.49335	0.020156
–	–	–	*CLDN7*	−0.49242	0.020437

Supplementary Table S1 (*Continued*)

Overexpressed genes			Underexpressed genes		
Name	Combined ES	*p*-Value	Name	Combined ES	*p*-Value
–	–	–	*NOD2*	−0.49217	0.020482
–	–	–	*DGKE*	−0.49214	0.020493
–	–	–	*LGMN*	−0.49015	0.021247
–	–	–	*MUC16*	−0.48956	0.021627
–	–	–	*RESF1*	−0.48942	0.02153
–	–	–	*GORASP2*	−0.48915	0.021577
–	–	–	*CD93*	−0.48896	0.045691
–	–	–	*MRNIP*	−0.48823	0.022044
–	–	–	*MORC4*	−0.48822	0.021917
–	–	–	*PLCXD1*	−0.48764	0.047602
–	–	–	*PSMB2*	−0.48723	0.022315
–	–	–	*PRIM2*	−0.48658	0.022687
–	–	–	*VPS37B*	−0.48645	0.022858
–	–	–	*WDR11*	−0.48639	0.029388
–	–	–	*CHN2*	−0.48542	0.023019
–	–	–	*NAGK*	−0.48536	0.023126
–	–	–	*ZNF200*	−0.48494	0.023293
–	–	–	*MAP2K7*	−0.48429	0.02336
–	–	–	*UNC119*	−0.48402	0.023707
–	–	–	*EPS8L1*	−0.48346	0.023722
–	–	–	*EPOR*	−0.48141	0.024575
–	–	–	*BSG*	−0.48135	0.039821
–	–	–	*LSR*	−0.48107	0.024838
–	–	–	*DDX46*	−0.48102	0.024725
–	–	–	*RAB40C*	−0.48067	0.024961
–	–	–	*GPR107*	−0.4799	0.025085
–	–	–	*CCDC6*	−0.4798	0.025152
–	–	–	*GAREM1*	−0.47815	0.025834
–	–	–	*POFUT2*	−0.47808	0.025979
–	–	–	*ADARB2*	−0.47791	0.026161
–	–	–	*NODAL*	−0.47788	0.025925
–	–	–	*GSDMD*	−0.47741	0.026237
–	–	–	*PPDPF*	−0.47721	0.026409
–	–	–	*ZNF177*	−0.47697	0.026237
–	–	–	*AKAP8L*	−0.47665	0.02653
–	–	–	*MKNK2*	−0.47601	0.027005
–	–	–	*CTBP1*	−0.4755	0.047811
–	–	–	*UPF2*	−0.47524	0.027113
–	–	–	*UGT2B28*	−0.47472	0.027286
–	–	–	*ZFP64*	−0.47466	0.027489
–	–	–	*C22orf46*	−0.47424	0.027454
–	–	–	*CBX1*	−0.47421	0.027454

(*Continued*)

Supplementary Table S1 (*Continued*)

Overexpressed genes			Underexpressed genes		
Name	Combined ES	*p*-Value	Name	Combined ES	*p*-Value
–	–	–	*DCAF8*	−0.47409	0.027734
–	–	–	*ALS2CL*	−0.47394	0.027563
–	–	–	*ENOSF1*	−0.47386	0.03336
–	–	–	*TREM1*	−0.4735	0.027734
–	–	–	*XPC*	−0.47262	0.028213
–	–	–	*TEC*	−0.47231	0.028538
–	–	–	*DDX28*	−0.472	0.028541
–	–	–	*CBLN1*	−0.47198	0.028434
–	–	–	*DDX39A*	−0.47106	0.028968
–	–	–	*COL13A1*	−0.47068	0.029195
–	–	–	*IFT122*	−0.46979	0.029388
–	–	–	*USP20*	−0.46946	0.029874
–	–	–	*MGAT5*	−0.46939	0.029603
–	–	–	*IL1B*	−0.4691	0.029816
–	–	–	*BCL10*	−0.46759	0.030478
–	–	–	*FAM118A*	−0.46749	0.03055
–	–	–	*MBTPS2*	−0.46748	0.030617
–	–	–	*ENO2*	−0.46698	0.030791
–	–	–	*SGPP1*	−0.46697	0.03079
–	–	–	*LATS1*	−0.46689	0.030994
–	–	–	*SLC10A3*	−0.46673	0.03104
–	–	–	*C19orf54*	−0.46544	0.031613
–	–	–	*KIAA0319*	−0.4652	0.031762
–	–	–	*ACAD10*	−0.46466	0.031887
–	–	–	*LRCH3*	−0.4646	0.031922
–	–	–	*DTX2P1-UPK3BP1-PMS2P11*	−0.46447	0.032282
–	–	–	*LINC01963*	−0.46444	0.032282
–	–	–	*OAS2*	−0.4639	0.03222
–	–	–	*DOLPP1*	−0.46307	0.03275
–	–	–	*ITK*	−0.46179	0.03336
–	–	–	*SEL1L*	−0.46175	0.03336
–	–	–	*PKD2L2*	−0.46166	0.033398
–	–	–	*ACOT11*	−0.46166	0.033405
–	–	–	*ARHGAP19*	−0.46123	0.033662
–	–	–	*NGFR*	−0.46119	0.033915
–	–	–	*ICE1*	−0.46097	0.033915
–	–	–	*PRR4*	−0.45982	0.034611
–	–	–	*PRAF2*	−0.4597	0.034438
–	–	–	*ANKRD27*	−0.45964	0.034441
–	–	–	*POLR2B*	−0.45952	0.034769
–	–	–	*TNFRSF1B*	−0.45949	0.034611
–	–	–	*EXOC3*	−0.45883	0.035034

Supplementary Table S1 (*Continued*)

Overexpressed genes			Underexpressed genes		
Name	Combined ES	*p*-Value	Name	Combined ES	*p*-Value
–	–	–	*WDR4*	−0.4586	0.034996
–	–	–	*ADGRA2*	−0.45726	0.035764
–	–	–	*POPDC3*	−0.45712	0.035928
–	–	–	*HMGXB3*	−0.4567	0.03608
–	–	–	*LRCH4*	−0.45666	0.036151
–	–	–	*PPP1R14D*	−0.45561	0.036637
–	–	–	*GRAMD4*	−0.45511	0.036995
–	–	–	*XDH*	−0.45498	0.037202
–	–	–	*PKP4*	−0.45471	0.037331
–	–	–	*RASA4*	−0.45448	0.037201
–	–	–	*ZNF804A*	−0.45444	0.037261
–	–	–	*APOH*	−0.45429	0.037331
–	–	–	*TMEM161A*	−0.45419	0.037331
–	–	–	*TNFRSF21*	−0.45414	0.037375
–	–	–	*FBXW4*	−0.45401	0.037764
–	–	–	*VEGFA*	−0.45282	0.038492
–	–	–	*DBR1*	−0.45135	0.039094
–	–	–	*ATPAF2*	−0.44981	0.040113
–	–	–	*QSER1*	−0.44916	0.040778
–	–	–	*PLEKHG6*	−0.44866	0.040859
–	–	–	*856*	−0.44769	0.041254
–	–	–	*BTN1A1*	−0.44652	0.042113
–	–	–	*FABP3*	−0.44611	0.042451
–	–	–	*TAS2R7*	−0.44588	0.042435
–	–	–	*DZANK1*	−0.44568	0.042859
–	–	–	*OCM2*	−0.44563	0.042781
–	–	–	*NDUFA10*	−0.44542	0.042767
–	–	–	*AK5*	−0.44535	0.042859
–	–	–	*HSD17B3*	−0.4451	0.042939
–	–	–	*SLC17A4*	−0.44337	0.044132
–	–	–	*CD9*	−0.44292	0.044381
–	–	–	*DDX47*	−0.44278	0.044532
–	–	–	*PTGES2*	−0.44266	0.044882
–	–	–	*GTF3C4*	−0.44194	0.04494
–	–	–	*FAM234B*	−0.4418	0.045037
–	–	–	*ECHDC2*	−0.44151	0.045137
–	–	–	*SMCHD1*	−0.44062	0.045691
–	–	–	*HEMK1*	−0.44029	0.046346
–	–	–	*MC1R*	−0.44016	0.04624
–	–	–	*DHX9*	−0.43831	0.047395
–	–	–	*AURKC*	−0.4382	0.047366
–	–	–	*ARHGAP33*	−0.43812	0.047395

(*Continued*)

Supplementary Table S1 (*Continued*)

Overexpressed genes			Underexpressed genes		
Name	Combined ES	*p*-Value	Name	Combined ES	*p*-Value
–	–	–	*SULT1A1*	−0.43784	0.047705
–	–	–	*COLEC12*	−0.43752	0.047809
–	–	–	*DRD5*	−0.43713	0.048457
–	–	–	*TBX21*	−0.4371	0.047983
–	–	–	*MAGEC2*	−0.43667	0.048314
–	–	–	*KHDRBS1*	−0.43648	0.048627
–	–	–	*PIGG*	−0.43647	0.048906
–	–	–	*CHST7*	−0.43564	0.049163

Abbreviations: DEG, differentially expressed gene; ES, effect size.
Note: The table is sorted on the basis of combined ES.

Supplementary Table S2 The complete list of 519 node genes of the zero-order network as obtained from Cytoscape using network analyzer

Gene name	Degree	Betweenness centrality	Closeness centrality	Combined ES	*p*-Value
TP53	60	0.294336	0.369736	−1.6588	0
UBB	39	0.202782	0.349528	0.48025	0.025064
HSP90AA1	31	0.193582	0.352381	0.70224	0.00034072
AKT1	30	0.133454	0.342366	−1.847	0.00022464
CDK1	27	0.052607	0.318769	0.65994	0.00089154
KAT2B	24	0.0692	0.32134	0.6207	0.0019535
PRPF8	23	0.033761	0.255172	−1.5438	0
SMAD3	22	0.075699	0.31318	−1.3522	5.3367E-12
CDC42	20	0.0827	0.289547	0.73897	0.0054548
HNRNPD	20	0.035921	0.27364	−0.82441	0.00031704
CSNK2A1	19	0.11239	0.315661	−0.51181	0.015109
DHX9	18	0.087967	0.290685	−0.43831	0.047395
RELA	18	0.080218	0.323144	−1.4588	2.1151E-13
RBX1	17	0.019157	0.272202	1.5674	0
PSMD4	16	0.002551	0.266324	0.80414	0.000031861
PSMC6	16	9.23E-04	0.267286	0.44812	0.041198
PSMB2	16	0.00169	0.267286	−0.48723	0.022315
HNRNPA3	16	0.001619	0.239482	−0.5779	0.0046098
SRSF4	16	0.005126	0.239593	−0.82971	0.00001668
PRKDC	16	0.055064	0.324765	−1.1922	6.3633E-10
SNRNP70	16	0.008639	0.239372	−1.3023	0.0016179
RPA1	16	0.034746	0.308885	−1.3668	0.022494
PSMA6	15	0.002367	0.270355	1.6761	5.18E-05
PSMA4	15	0.001262	0.267148	0.87204	0.000012304
PSMA1	15	0.001262	0.267148	0.57491	0.0048252
PSMD11	15	0.001	0.266598	−0.60114	0.0028868
PRPF6	15	0.004138	0.237179	−1.4164	0.001028
PSMD1	14	7.07E-04	0.26701	0.44564	0.042767

Supplementary Table S2 (*Continued*)

Gene name	Degree	Betweenness centrality	Closeness centrality	Combined ES	*p*-Value
PTPN11	14	0.041867	0.275239	−0.54241	0.0087296
TBP	14	0.0272	0.294989	−1.3206	1.4101E-11
PIK3CB	13	0.034451	0.270214	1.045	5.1571E-08
PSMD6	13	0.002828	0.270921	0.61953	0.0020103
UBE2E1	13	0.004712	0.287938	0.48864	0.022027
KIT	13	0.026996	0.271488	−0.69175	0.00043412
FOS	13	0.02741	0.305605	−1.028	8.581E-08
UBE2I	13	0.023049	0.284303	−1.1682	1.2814E-09
ABL1	13	0.029396	0.312425	−1.2514	1.0793E-10
IKBKB	13	0.019905	0.307418	−1.252	1.0711E-10
SUMO2	12	0.010419	0.28603	1.0784	0.030994
RPL26	12	0.021925	0.280607	0.83795	0.0080585
UCHL5	12	0.001947	0.26701	0.57507	0.0048178
POLR2B	12	0.015032	0.251212	−0.45952	0.034769
EEF2	12	0.041472	0.284772	−0.64594	0.0011563
CDK9	12	0.033765	0.290685	−0.74094	0.00014496
SNRPB	12	0.012916	0.236854	−0.73274	0.00017165
SHC1	12	0.018312	0.278195	−1.0818	0.024837
HNRNPU	12	0.007573	0.241379	−1.2189	0.012529
SF3A1	12	8.96E-04	0.23827	−1.3026	2.4561E-11
JAK1	12	0.016309	0.274801	−1.5369	0.00012552
GSK3B	11	0.012614	0.304169	0.82695	0.02061
HNRNPH1	11	0.00738	0.234495	−0.60898	0.0024714
RNPS1	11	0.007147	0.240818	−1.3984	1.1706E-12
RPL31	10	0.002576	0.247255	1.3187	1.41E-11
RPL38	10	0.002576	0.247255	0.43774	0.047983
PML	10	0.010331	0.293817	−0.51123	0.015122
YY1	10	0.010659	0.290196	−0.5429	0.0086686
DDB1	10	0.013285	0.279396	−0.67381	0.00064692
TYK2	10	0.016016	0.263078	−0.86106	7.5829E-06
POLR2E	10	0.014431	0.248322	−0.85476	0.024425
PRPF19	10	0.005148	0.239372	−1.0503	4.5269E-08
KAT5	10	0.008716	0.288098	−1.0494	4.4696E-08
AKT2	10	0.025373	0.295157	−1.3094	0.0091885
RPL27	9	0.001302	0.247137	1.5836	1.61E-07
NEDD8	9	0.006072	0.280911	0.92641	1.3403E-06
POLR2J	9	0.011512	0.241155	0.6755	0.011392
XPC	9	0.006695	0.279849	−0.47262	0.028213
SNRNP40	9	1.64E-04	0.224242	−0.50621	0.016318
STX4	9	0.014641	0.203536	−0.50526	0.01652
POLE	9	0.003358	0.260957	−0.73772	0.00015187
RBBP4	9	0.010261	0.269091	−0.93717	1.0243E-06
POLA1	9	0.00412	0.25669	−1.0201	0.0014592

(*Continued*)

Supplementary Table S2 (*Continued*)

Gene name	Degree	Betweenness centrality	Closeness centrality	Combined ES	*p*-Value
EHMT2	9	0.012217	0.279245	−1.1042	9.1468E-09
MCM3	9	0.011408	0.270637	−1.1951	5.6106E-10
RAF1	9	0.011062	0.282289	−1.2037	4.65E-10
PSME3	9	4.90E-04	0.241155	−1.5757	0
HIST3H3	9	0.008503	0.25555	1.0238	0
RPL39	8	0.00104	0.232704	1.2694	0.00033825
INTS6	8	0.005769	0.213608	0.7977	0.000037578
UBE2N	8	0.012324	0.263882	0.67193	0.00066762
POLR2L	8	0.001249	0.213696	0.61211	0.0023313
CCNB1	8	0.00511	0.28306	0.48227	0.02421
NCOA3	8	0.004718	0.294653	0.46785	0.030683
ADRM1	8	0	0.220801	−0.55524	0.0069779
SNW1	8	0.013333	0.256436	−0.60534	0.0026316
INTS5	8	0.005769	0.213608	−0.78341	0.000051748
PLCB2	8	0.013401	0.224048	−0.80358	0.000031948
NCOR2	8	0.008864	0.284615	−0.97801	3.4888E-07
DNMT1	8	0.016454	0.291667	−1.0301	0.033548
DYNC1H1	8	0.026766	0.2677	−1.1255	4.7494E-09
PTBP1	8	0.005358	0.234389	−1.7024	1.8342E-09
TP53BP1	8	0.005874	0.28337	−1.7142	0
RSRC1	7	1.29E-04	0.220145	1.4612	1.52E-13
PSMD9	7	0	0.260302	0.9907	2.4439E-07
SEC61G	7	0.011235	0.264286	0.91734	1.7777E-06
SNAP29	7	0.002343	0.198696	0.77084	0.000070752
NRAS	7	0.002966	0.245149	0.71886	0.0023826
STX3	7	0.003312	0.198315	−0.67073	0.00069289
SUPT5H	7	0.006167	0.238161	−0.69212	0.0027714
ERCC3	7	0.008965	0.290848	−0.72193	0.00022002
MFAP1	7	4.77E-04	0.217647	−0.77392	0.000064471
EIF4G1	7	0.030893	0.277302	−0.84295	0.000012364
NCOA1	7	0.00434	0.258225	−0.96575	4.7767E-07
DDX23	7	2.00E-06	0.224145	−0.96994	0.018318
NXF1	7	0.028614	0.233228	−0.99059	2.4008E-07
TSC1	7	0.00137	0.27364	−1.0078	1.4952E-07
FUS	7	6.40E-05	0.219306	−1.1572	1.7906E-09
MCM7	7	0.002601	0.266873	−1.2676	0.015775
HNRNPA0	7	0.002768	0.234601	−1.6186	0
STX8	6	0.004824	0.198467	1.288	3.71E-11
MRPL15	6	4.16E-04	0.236962	1.2359	1.77E-10
BARD1	6	0.004521	0.283836	1.084	1.6405E-08
RPS9	6	2.58E-04	0.22561	1.0567	3.7345E-08
KRAS	6	0.002226	0.242056	1.0177	1.1211E-07
NBN	6	0.001533	0.240818	0.97705	3.5642E-07

Supplementary Table S2 (*Continued*)

Gene name	Degree	Betweenness centrality	Closeness centrality	Combined ES	*p*-Value
TUBG1	6	0.011501	0.267148	0.95932	5.7656E-07
ARHGEF12	6	0.012951	0.235027	0.69173	0.00043871
VAMP4	6	0.031735	0.244455	0.67772	0.00058391
RPL36AL	6	0	0.219864	0.66031	0.00087697
KHDRBS1	6	0.014547	0.266873	−0.43648	0.048627
VCP	6	0.01635	0.259779	−0.4962	0.019416
RAD52	6	0.001918	0.259	−0.52975	0.010924
SQSTM1	6	0.007078	0.279698	−0.56311	0.006104
MRPL4	6	4.16E-04	0.236962	−0.57848	0.0045643
CASP8	6	0.017997	0.278495	−0.6345	0.0014592
VDR	6	0.002806	0.28603	−0.64541	0.039164
ACACB	6	0.034312	0.175772	−0.71956	0.00023183
AXIN1	6	0.013883	0.284303	−0.86133	7.5829E-06
CHD3	6	0.004232	0.2677	−1.0324	7.3645E-08
PARP1	6	0.005134	0.282289	−1.2416	1.4287E-10
RHOBTB2	6	0.008946	0.265914	−1.2866	3.9071E-11
VAMP2	6	0.004314	0.19171	−1.6098	0
UBE4B	6	0.003306	0.240037	−1.6141	0
GTF3A	5	0.001828	0.247255	1.5392	0
SNCA	5	0.014051	0.246432	−1.6815	0.048833
NEDD4	5	0.002506	0.262278	0.91717	1.7399E-06
NRIP1	5	0.004035	0.246432	0.93616	0.0027229
GRAP2	5	0.004369	0.22512	0.65454	0.00097273
AKT3	5	0.002256	0.272775	0.58252	0.0041851
ERBB4	5	0.003329	0.230325	0.44513	0.042939
CTBP1	5	0.004201	0.247728	−0.4755	0.047811
POLA2	5	0	0.24026	−0.5482	0.007917
ERCC5	5	0.0011	0.253922	−0.56633	0.0057042
HNRNPDL	5	8.38E-04	0.215833	−0.65928	0.00089076
PPP4C	5	0.011639	0.255424	−0.65746	0.00091284
MAPK7	5	0.005025	0.278195	−0.69762	0.0011694
SUGP1	5	0.038296	0.23871	−0.69201	0.019934
MED17	5	0.00482	0.207532	−0.72473	0.00020609
PABPN1	5	0	0.232496	−0.78057	0.000055179
JUND	5	0.005062	0.250121	−0.84285	0.000012469
TRIM28	5	0.007037	0.275532	−0.9264	1.3495E-06
IRF1	5	0.008904	0.265097	−0.93284	0.011317
TSC2	5	7.67E-05	0.265369	−0.94142	9.1621E-07
SETD2	5	0.005422	0.279849	−0.98617	2.7233E-07
SSRP1	5	0.006178	0.252314	−1.0567	3.6183E-08
DNMT3A	5	0.004289	0.243536	−1.1477	2.3684E-09
PAK1	5	0.003025	0.283215	−1.1802	0.034438
MEF2D	5	0.009037	0.275093	−1.2053	0.0066041

(*Continued*)

Supplementary Table S2 (*Continued*)

Gene name	Degree	Betweenness centrality	Closeness centrality	Combined ES	*p*-Value
ANAPC1	5	0.004129	0.267286	−1.3647	0.000049786
NUP93	5	0.005792	0.189744	−2.3158	0.01051
SP100	4	2.20E-04	0.241604	1.3322	2.44E-05
POMP	4	0	0.213344	1.2768	3.27E-05
LSM6	4	0	0.203937	1.1202	5.65E-09
POLR1D	4	1.19E-04	0.203058	1.0948	6.79E-07
RNF11	4	0.002856	0.26004	1.0389	0.015332
CKS1B	4	4.46E-04	0.252314	0.95972	5.92E-07
GTF2E2	4	0.001389	0.245614	0.95478	6.8255E-07
KAT6A	4	0.004652	0.283215	0.57597	0.0047292
CDC34	4	7.48E-04	0.241717	0.55945	0.0065143
RALGDS	4	0.00107	0.23893	0.53988	0.0091885
SCO2	4	0.003887	0.1935	0.52108	0.012897
BIRC3	4	0.005784	0.218381	0.44643	0.042289
LSM2	4	0	0.203937	0.43988	0.04624
ATP6V1E1	4	0.007715	0.17284	0.44215	0.044857
VEGFA	4	0.004193	0.278345	−0.45282	0.038492
OAS2	4	0.0049	0.266598	−0.4639	0.03222
EXOC3	4	0.010268	0.231871	−0.45883	0.035034
CBX1	4	0.003947	0.225315	−0.47421	0.027454
EPOR	4	4.02E-04	0.235134	−0.48141	0.024575
HTT	4	0.007188	0.287938	−0.55288	0.016927
MSH6	4	4.99E-04	0.281522	−0.55339	0.016128
UIMC1	4	8.06E-05	0.274801	−0.58	0.0044664
USP34	4	0.023472	0.262146	−0.6368	0.0014153
PEX14	4	0.007707	0.140303	−0.6506	0.0010548
TGFBR2	4	0.005533	0.257455	−0.70602	0.00031755
KHSRP	4	0.009929	0.269792	−0.74116	0.00014029
SYNJ1	4	2.49E-06	0.215743	−0.76943	0.00025436
POLR3E	4	1.19E-04	0.203058	−0.79521	0.000038915
MED15	4	0.008227	0.243192	−0.86695	0.010593
NUP98	4	0.009619	0.189744	−0.87775	0.040951
CS	4	0.009727	0.150014	−0.88656	3.8587E-06
AGO1	4	0.00407	0.287618	−0.91619	0.000001787
EHMT1	4	3.43E-04	0.299076	−0.94505	8.2593E-07
ZC3H13	4	0.002984	0.233333	−0.97369	0.0025115
MAVS	4	5.60E-04	0.264962	−1.0036	1.7317E-07
RANGAP1	4	0.009543	0.236962	−1.0492	2.8429E-06
PRDM1	4	7.08E-04	0.231871	−1.0702	2.5999E-08
PACS1	4	0.026758	0.243421	−1.0879	1.5229E-08
RBM22	4	7.47E-06	0.203696	−1.0775	1.9848E-08
HGS	4	0.006845	0.260171	−1.1095	0.010177
DVL2	4	0.004516	0.251701	−1.1674	1.3617E-09

Supplementary Table S2 (*Continued*)

Gene name	Degree	Betweenness centrality	Closeness centrality	Combined ES	*p*-Value
SGK1	4	7.50E-04	0.278794	−1.2508	0.00004597
GTF3C2	4	0.002077	0.228799	−1.2506	1.0808E-10
ELAVL1	4	0.003857	0.276709	−1.6071	0
SIN3B	4	0.004621	0.278195	−1.6951	0
EP400	4	0.001232	0.272919	−1.7961	0
PEX5	4	0.019186	0.162893	−1.8499	0
IL6ST	3	0	0.235027	1.4314	0.002628
UBL5	3	3.55E-04	0.203616	1.4342	5.72E-05
RSL24D1	3	0	0.198544	1.2732	5.73E-11
VAMP5	3	5.66E-06	0.169336	1.2216	2.53E-10
OPTN	3	0.001097	0.233649	1.0869	1.52E-08
TNFAIP3	3	0.003913	0.236854	1.029	2.39E-07
CHMP4A	3	0.006835	0.207449	1.0621	3.23E-08
SOD1	3	0.002896	0.256182	1.0579	3.69E-08
ARPC2	3	0.007715	0.184145	0.92102	4.19E-05
STAT4	3	2.27E-04	0.223084	0.87978	4.5927E-06
DYNLT1	3	0.007707	0.211688	0.85877	8.0251E-06
PPP2R5C	3	0.005253	0.288258	0.85015	0.000010436
DYNC1LI2	3	1.87E-05	0.211688	0.83993	0.000012897
HOXA10	3	0.007715	0.216284	0.80165	0.000032952
DYNC1I2	3	3.73E-05	0.211601	0.79552	0.000038314
CHMP5	3	0.014392	0.260302	0.7733	0.000065407
PRDX1	3	0.001522	0.241042	0.77298	0.0028273
TRIB3	3	4.04E-04	0.268812	0.69526	0.00040622
MYO6	3	0.007752	0.227692	0.67788	0.00058985
RBBP8	3	2.86E-04	0.229712	0.59674	0.035423
SCP2	3	0.022942	0.19379	0.53114	0.010716
MRPL12	3	0	0.219492	0.51587	0.013961
ANXA2	3	0.00791	0.282751	0.5032	0.01719
BIRC2	3	0.001923	0.218289	0.46221	0.033213
SERBP1	3	6.59E-04	0.238051	−1.7961	0.049401
CD9	3	0.00467	0.214138	−0.44292	0.044381
GTF3C4	3	0.001797	0.228194	−0.44194	0.04494
ITK	3	0.002758	0.234921	−0.46179	0.03336
NGFR	3	2.46E-04	0.246667	−0.46119	0.033915
NFKBIB	3	1.42E-04	0.256944	−0.51422	0.014416
ARNTL	3	0.003899	0.224924	−0.52522	0.04733
MYO5A	3	0.003363	0.229916	−0.52998	0.01091
VAMP3	3	5.66E-06	0.169392	−0.55291	0.0073067
MDH2	3	0.003891	0.149711	−0.55226	0.0073519
HNRNPH3	3	2.49E-06	0.190301	−0.59421	0.0033023
HNRNPUL1	3	4.85E-05	0.196212	−0.6125	0.0023122
ELP3	3	3.64E-04	0.24856	−0.61561	0.0021859

(*Continued*)

Supplementary Table S2 (*Continued*)

Gene name	Degree	Betweenness centrality	Closeness centrality	Combined ES	*p*-Value
PIP5K1C	3	0	0.215654	−0.62126	0.0019508
TFIP11	3	0.001912	0.204662	−0.6265	0.0017435
RAB5A	3	0.004884	0.171127	−0.64776	0.0011291
PIP4K2C	3	0	0.215654	−0.65308	0.0011605
VAMP1	3	5.66E-06	0.169336	−0.68276	0.00091009
DROSHA	3	0.001549	0.277599	−0.68995	0.00046018
ATP6V1B2	3	0.015354	0.208451	−0.77413	0.000065743
AP1B1	3	0.003861	0.12425	−0.78311	0.000053426
MYO9A	3	3.67E-05	0.234283	−0.79474	0.000039489
CLINT1	3	0.011538	0.141724	−0.81855	0.0065978
NFYA	3	4.58E-04	0.281981	−0.81722	0.00002269
SMARCD1	3	4.63E-04	0.282906	−0.84239	0.000012304
CAD	3	0.049073	0.211429	−0.87341	5.4602E-06
TUBGCP3	3	1.87E-05	0.211256	−0.88387	4.2711E-06
SNAPC4	3	0.002489	0.229712	−0.89518	3.0437E-06
CDH2	3	0.007707	0.221842	−0.92466	1.4055E-06
SENP3	3	0.007707	0.273784	−0.93299	1.1299E-06
HMOX1	3	0.003987	0.26122	−0.94448	8.5172E-07
USP11	3	0.001473	0.271773	−0.99161	2.3933E-07
GRAP	3	0.001285	0.233966	−1.031	4.7476E-07
CXCR4	3	0.007715	0.216284	−1.0255	9.0597E-08
TRIO	3	4.04E-05	0.234177	−1.0847	1.635E-08
IRF3	3	8.03E-04	0.276414	−1.0854	8.8891E-08
TAF6	3	0	0.245614	−1.1705	1.2182E-09
RHOT2	3	9.13E-04	0.234071	−1.1729	1.134E-09
ILF3	3	8.83E-04	0.261748	−1.1255	0.0023117
NEDD9	3	0.002657	0.263479	−1.2218	2.5346E-10
LEF1	3	0.015377	0.239482	−1.326	3.7056E-11
TAF4	3	0	0.245614	−1.5439	0
TCEB1	3	1.21E-04	0.262944	−1.4357	0
UFD1L	3	2.61E-04	0.222222	1.4391	0
TNPO1	2	0.003861	0.15963	1.6642	0.0060162
CRBN	2	0	0.232704	1.4826	8.2947E-14
RNF7	2	5.56E-04	0.243307	1.2653	7.1245E-11
NLK	2	0.007707	0.193573	1.0638	2.9636E-08
ACAT1	2	0.001908	0.149624	1.0257	9.1321E-08
XRCC4	2	1.91E-04	0.246902	0.95046	7.2308E-07
ATP6V1D	2	0	0.172724	0.94073	9.2531E-07
RGCC	2	0.001361	0.258741	0.92522	1.3755E-06
PRDX2	2	0	0.20904	0.87716	4.8851E-06
RPA4	2	0	0.23893	0.89347	0.01964
SIAH2	2	9.20E-04	0.228194	0.88423	0.021393
ABCA1	2	0.007707	0.225022	0.79253	0.000042033

Supplementary Table S2 (*Continued*)

Gene name	Degree	Betweenness centrality	Closeness centrality	Combined ES	*p*-Value
F2R	2	1.05E-04	0.175	0.75827	0.000093994
PEX2	2	0	0.140227	0.7432	0.00013442
ABCD3	2	0	0.123099	0.69195	0.00043428
HAT1	2	0	0.223469	0.68919	0.0004566
SOCS5	2	0	0.222988	0.62363	0.0018387
PLA2G4A	2	4.18E-04	0.23838	0.61988	0.0019891
GATA6	2	8.65E-04	0.226003	0.581	0.0042788
NPEPPS	2	0	0.259519	0.56395	0.0059909
RIDA	2	0	0.192924	0.56183	0.0062
TRIP4	2	0	0.198544	0.5465	0.0081475
PEX3	2	0	0.123099	0.53993	0.0092023
GINS2	2	0	0.213169	0.50412	0.016873
MKKS	2	0.052695	0.264421	0.49814	0.018627
REL	2	0	0.244455	0.48116	0.024627
MRGBP	2	0	0.223855	0.47808	0.025973
GNA12	2	9.62E-04	0.190933	0.47234	0.028367
PRKAR2B	2	0	0.265233	−1.1418	0.032289
HBEGF	2	4.73E-05	0.187885	0.46227	0.033213
AURKC	2	1.70E-04	0.229204	−0.4382	0.047366
SEL1L	2	0.003861	0.206457	−0.46175	0.03336
WRAP53	2	0	0.218842	−2.0266	0.049722
TEC	2	0	0.213873	−0.47231	0.028538
UPF2	2	0	0.222032	−0.47524	0.027113
CHN2	2	3.67E-05	0.233649	−0.48542	0.023019
GOT2	2	0.007558	0.175059	−0.49923	0.018306
G3BP1	2	0.003861	0.217373	−0.5016	0.017577
FOSB	2	1.88E-04	0.224048	−0.51313	0.01466
NSFL1C	2	0	0.214315	−0.51586	0.013941
AHCTF1	2	0	0.159679	−0.52162	0.01264
CDC25B	2	0	0.247255	−0.52353	0.012204
HEY1	2	9.82E-05	0.202028	−0.53128	0.010705
TEX10	2	0.003861	0.215206	−0.53704	0.0096283
SPIB	2	6.52E-04	0.24183	−0.55556	0.0069913
UBE2M	2	0	0.231044	−0.55614	0.0068624
PLXNB2	2	0.003861	0.190511	−0.57191	0.0051274
SARS2	2	0	0.181691	−0.58557	0.0039346
IDH3A	2	0.003861	0.130544	−0.58841	0.003693
TRAK1	2	2.85E-05	0.210655	−0.58925	0.0036569
POLD2	2	0	0.213696	−0.62181	0.0025421
BPTF	2	6.01E-05	0.212295	−0.65821	0.00096211
C7orf26	2	0	0.176131	−0.65265	0.0010109
AP1G1	2	0	0.124221	−0.6846	0.0002021
BACH2	2	1.67E-04	0.223469	−0.7537	0.008215

(*Continued*)

Supplementary Table S2 (*Continued*)

Gene name	Degree	Betweenness centrality	Closeness centrality	Combined ES	*p*-Value
PVR	2	0.003861	0.174882	−0.7407	0.00014454
RNF40	2	3.08E-04	0.2326	−0.75843	0.000094069
DET1	2	0	0.232704	−0.73821	0.0025127
NUP205	2	0	0.189397	−0.77798	0.000058791
PCNT	2	0	0.211084	−0.78148	0.000054008
ACO2	2	1.87E-05	0.130544	−0.80678	0.0072417
INTS9	2	0	0.176131	−0.81261	0.000025667
GATA2	2	0.001265	0.25991	−0.82349	0.00001957
IL12RB1	2	0	0.208451	−0.83477	0.000014739
XRCC1	2	2.49E-04	0.242056	−0.87411	5.4575E-06
MEN1	2	0	0.23893	−0.89103	3.3896E-06
SNIP1	2	3.32E-04	0.20442	−0.8929	3.2185E-06
ZNF592	2	0	0.176131	−0.90735	2.2209E-06
CD3E	2	0	0.228496	−0.91929	0.0052006
NR1H2	2	0.003861	0.183884	−0.92075	1.5532E-06
MDC1	2	0	0.270921	−0.93569	1.0779E-06
MMS19	2	6.50E-05	0.235669	−0.95337	7.0589E-07
CXXC1	2	0.003861	0.226102	−0.9657	4.9724E-07
RBM5	2	0	0.203456	−0.97037	4.2755E-07
GEMIN4	2	0.003861	0.19171	−0.97157	4.2311E-07
WIPF1	2	0.011538	0.225217	−1.01	1.4535E-07
GTF3C1	2	0	0.186331	−1.0199	1.0809E-07
SUPT6H	2	0	0.206869	−1.0522	0.00020749
PPIF	2	0.01926	0.278195	−1.0642	2.9495E-08
APBB1	2	9.61E-05	0.244917	−1.0718	0.000000025
MCM3AP	2	0.003861	0.213256	−1.0548	3.7666E-08
SRRM2	2	0	0.193717	−1.1417	2.9572E-09
GGA2	2	0.015354	0.164758	−1.1084	8.1115E-09
ACTN4	2	7.81E-04	0.162382	−1.1513	2.1328E-09
TCF7	2	0.003861	0.162332	−1.1433	2.8041E-09
RPAP1	2	0	0.194664	−1.1489	2.2773E-09
CUL7	2	4.57E-04	0.272775	−1.115	0.017324
PDLIM2	2	0.003654	0.188022	−1.1972	5.381E-10
ZMIZ1	2	4.73E-04	0.245964	−1.247	0.00018511
RNF4	2	0	0.23156	−1.2083	3.9398E-10
USP19	2	0	0.179363	−1.2879	3.9071E-11
IGF2R	2	0.019156	0.196584	−1.3442	0.000000256
MAZ	2	7.80E-04	0.24026	−1.3608	4.0938E-12
CABIN1	2	0.003861	0.216013	−1.3579	4.387E-12
BHLHE40	2	0.00453	0.270779	−1.4408	0.0010802
CTCF	2	0.003861	0.23882	−1.448	1.0305E-12
INPP5B	2	3.96E-04	0.214138	−1.6033	0
PRKAG2	2	0.003861	0.149624	−1.6726	0.014448

Supplementary Table S2 (*Continued*)

Gene name	Degree	Betweenness centrality	Closeness centrality	Combined ES	*p*-Value
PRKCDBP	2	0	0.181691	1.2045	0
BATF	1	0	0.200155	1.9152	0.016655
IGFBP7	1	0	0.21783	1.6631	0.00031067
LGALS1	1	0	0.16092	1.5378	8.29E-14
DCAF10	1	0	0.218473	1.4322	4.2003E-13
SEC62	1	0	0.209124	1.3925	0.044882
ANAPC15	1	0	0.210998	1.2139	0.0045486
ATP6V0E1	1	0	0.14741	1.3675	0.00043622
TPT1	1	0	0.270073	1.2123	3.4413E-10
SUMO4	1	0	0.221462	1.163	0.0018799
BLVRB	1	0	0.2072	1.1214	0.012816
ELL2	1	0	0.225315	1.0064	0.0072417
PERP	1	0	0.270073	0.92132	1.5322E-06
DCAF6	1	0	0.218473	0.93505	0.0027032
ACAT2	1	0	0.149538	0.88533	0.0056612
ARPC5L	1	0	0.155556	0.85631	8.4673E-06
TANK	1	0	0.235241	0.83771	0.0041407
ETHE1	1	0	0.270073	0.80201	0.0013871
ATF6	1	0	0.225022	0.80705	0.000029741
NAPG	1	0	0.165654	0.77572	0.00006303
PTPN2	1	0	0.215654	0.76259	0.010493
PPP4R4	1	0	0.203536	0.71095	0.00028187
LEPR	1	0	0.215923	0.70464	0.00032292
GMFG	1	0	0.155556	0.68839	0.00046468
CDC42BPA	1	0	0.224631	0.67303	0.0038764
PTGFR	1	0	0.183104	0.67299	0.00066016
DNAJC6	1	0	0.110542	0.67234	0.00066004
KLF5	1	0	0.233333	0.66576	0.00076863
DNAJA1	1	0	0.260695	0.65812	0.00091273
FNBP1L	1	0	0.224631	0.65539	0.00095691
DUSP4	1	0	0.217739	0.64822	0.0011068
PBX1	1	0	0.177885	0.65276	0.0010084
CREM	1	0	0.234177	0.64507	0.0011797
SNUPN	1	0	0.191568	−1.1941	0.034799
TCF4	1	0	0.193284	0.63061	0.0015864
SDC4	1	0	0.177885	0.62773	0.0017018
CD226	1	0	0.148893	0.61523	0.0021891
PIN4	1	0	0.162179	0.60488	0.0026591
ABCA12	1	0	0.155369	0.59015	0.0035638
RAP1GAP	1	0	0.24183	0.59765	0.0031086
CETN3	1	0	0.175832	0.58363	0.0041002
TNIP3	1	0	0.191568	0.55381	0.0071809
ME1	1	0	0.130249	0.55188	0.0074569

(*Continued*)

Supplementary Table S2 (*Continued*)

Gene name	Degree	Betweenness centrality	Closeness centrality	Combined ES	*p*-Value
ENC1	1	0	0.21405	0.55505	0.028781
MYCN	1	0	0.240037	0.54006	0.0091932
PARD6B	1	0	0.224631	0.52942	0.01097
HELLS	1	0	0.225905	0.52025	0.013087
PAQR3	1	0	0.220238	0.51331	0.014625
FGFR3	1	0	0.260695	0.49418	0.019895
DYNC2LI1	1	0	0.211256	0.49827	0.018592
GMPS	1	0	0.130183	0.49035	0.021394
SCFD1	1	0	0.16092	0.48062	0.025064
S100A10	1	0	0.220519	0.46446	0.031927
BAG1	1	0	0.220238	0.45362	0.037725
TGM2	1	0	0.223661	0.45414	0.037331
MAGEC2	1	0	0.216103	−0.43667	0.048314
SULT1A1	1	0	0.207782	−0.43784	0.047705
PKP4	1	0	0.224631	−0.45471	0.037331
AKAP8L	1	0	0.225315	−0.47665	0.02653
MKNK2	1	0	0.217191	−0.47601	0.027005
CCDC6	1	0	0.203536	−0.4798	0.025152
NODAL	1	0	0.2386	−0.47788	0.025925
MEPCE	1	0	0.225315	−0.50696	0.016167
KLK6	1	0	0.197785	−0.50939	0.015728
HDLBP	1	0	0.192852	−0.51904	0.013225
GORASP1	1	0	0.24183	−0.53555	0.010029
IRF8	1	0	0.209632	−0.53582	0.0098595
IRF9	1	0	0.209632	−0.54159	0.0088838
PELI2	1	0	0.208871	−0.56047	0.0063329
GNAT2	1	0	0.183104	−0.57113	0.005252
UBN1	1	0	0.177702	−0.59061	0.0035396
PPP4R1	1	0	0.203536	−0.60652	0.0026087
FOXH1	1	0	0.2386	−0.6058	0.0026101
RNF185	1	0	0.201165	−0.6082	0.0025127
CXCR5	1	0	0.177885	−0.63416	0.0014794
PPP1R13B	1	0	0.270073	−0.63118	0.0015725
ESRRA	1	0	0.197785	−0.6342	0.0014684
PPP6R1	1	0	0.245265	−0.64783	0.0011208
EMSY	1	0	0.183949	−0.66207	0.00083189
FXR2	1	0	0.223469	−0.65932	0.00088769
YEATS2	1	0	0.243307	−0.66908	0.00070899
IPO4	1	0	0.137693	−0.67681	0.00059354
MX2	1	0	0.210569	−0.68194	0.00053157
HUWE1	1	0	0.191639	−0.68314	0.00052476
OTUB1	1	0	0.208871	−0.68686	0.00048407
NUP210	1	0	0.159532	−0.68708	0.00047532

Supplementary Table S2 (*Continued*)

Gene name	Degree	Betweenness centrality	Closeness centrality	Combined ES	*p*-Value
PTPRO	1	0	0.213608	−0.69236	0.016224
MED22	1	0	0.171922	−0.71094	0.00028408
ENG	1	0	0.204824	−0.71675	0.0002517
PACS2	1	0	0.179301	−0.72328	0.00021903
FGFR1OP	1	0	0.195841	−0.71484	0.025064
IDH3G	1	0	0.115496	−0.72744	0.00019434
INPP5E	1	0	0.212818	−0.7438	0.00013289
CHFR	1	0	0.220238	−0.74284	0.00013501
PPARD	1	0	0.260695	−0.76847	0.000074971
SCARB1	1	0	0.140114	−0.75788	0.011035
ZNF76	1	0	0.227893	−0.7965	0.000038137
PITPNM1	1	0	0.24183	−0.80265	0.000032518
EDEM1	1	0	0.171183	−0.81181	0.000026276
CC2D1A	1	0	0.171865	−0.81048	0.000027244
DGKZ	1	0	0.183104	−0.82026	0.000021133
PTAFR	1	0	0.208367	−0.82301	0.00002019
FNBP4	1	0	0.21074	−0.8329	0.000015476
AACS	1	0	0.149538	−0.8012	0.01442
CRY2	1	0	0.183688	−0.81292	0.0049588
RABEP2	1	0	0.146163	−0.8466	0.000011481
ZMIZ2	1	0	0.221462	−0.91276	0.00010805
TFCP2	1	0	0.225022	−0.8828	0.044875
ASPSCR1	1	0	0.206292	−0.88559	3.9114E-06
ITGA5	1	0	0.176431	−0.88704	3.8012E-06
CBFA2T3	1	0	0.217739	−0.89583	3.0181E-06
GTF2H4	1	0	0.225413	−0.90883	2.1905E-06
TOMM34	1	0	0.260695	−0.91644	1.7775E-06
HNRNPAB	1	0	0.189952	−0.91206	0.045276
MDN1	1	0	0.177155	−0.94765	0.000047558
E4F1	1	0	0.270073	−0.88414	0.0030425
ZBTB18	1	0	0.195915	−0.98085	0.0019429
ATP6V1G2	1	0	0.14741	−0.9597	0.03317
TELO2	1	0	0.245265	−0.96687	4.7329E-07
CAPRIN1	1	0	0.178621	−0.98743	0.0078046
LUC7L2	1	0	0.193211	−0.98237	3.0245E-07
CHMP1B	1	0	0.206621	−1.0027	1.7317E-07
PAXIP1	1	0	0.220896	−0.99824	1.962E-07
KLHL3	1	0	0.21405	−0.9927	2.3149E-07
CEP72	1	0	0.210912	−0.99385	0.000037924
PILRA	1	0	0.215923	−1.0306	7.6599E-08
GRK5	1	0	0.197785	−0.99384	2.2674E-07
RFX1	1	0	0.238161	−1.0584	3.525E-08
PRPF31	1	0	0.203377	−1.0562	3.6415E-08

(*Continued*)

Supplementary Table S2 (*Continued*)

Gene name	Degree	Betweenness centrality	Closeness centrality	Combined ES	*p*-Value
LUC7L	1	0	0.193211	−1.0698	0.0012958
ACTR1B	1	0	0.211256	−1.0897	1.4829E-08
BRD1	1	0	0.220801	−1.1399	3.0065E-09
PARN	1	0	0.212556	−1.1284	4.3071E-09
PBX2	1	0	0.177885	−1.1986	5.3558E-10
WWP2	1	0	0.2386	−1.2055	4.3343E-10
TLE3	1	0	0.139698	−1.2165	2.9961E-10
DNAJA3	1	0	0.260695	−1.2453	1.307E-10
SH2B3	1	0	0.213608	−1.2899	3.6125E-11
BANP	1	0	0.270073	−1.2401	0.000084578
VPS37C	1	0	0.206539	−1.368	3.19E-12
NELFA	1	0	0.192422	−1.3751	2.6223E-12
SETD1B	1	0	0.184473	−1.5485	2.8202E-12
NOLC1	1	0	0.240037	−1.639	9.9536E-13
MAP4K2	1	0	0.224631	−1.7048	4.2798E-06
TACC1	1	0	0.160074	−1.725	1.134E-09
HIST1H4F	1	0	0.203616	−1.2048	4.3271E-09
FYB	1	0	0.183818	0.61766	0.0021035

Abbreviation: ES, effect size.
Note: The expression level (combined ES) and *p*-value of the genes were added from the meta-analysis in the table.
